# Development of an evidence-based decision aid on complementary and alternative medicine (CAM) and pain for parents of children with cancer

**DOI:** 10.1007/s00520-019-05058-8

**Published:** 2019-09-06

**Authors:** Miek C. Jong, Inge Boers, Herman van Wietmarschen, Martine Busch, Marianne C. Naafs, Gertjan J. L. Kaspers, Wim J. E. Tissing

**Affiliations:** 1 Department of Health Sciences, Mid Sweden University, Holmgatan 10, 851 70 Sundsvall, Sweden; 2 Department Nutrition & Health, Louis Bolk Institute, Kosterijland 3-5, 3981 AJ Bunnik, The Netherlands; 3 Van Praag Institute, Springweg 7, 3511 VH Utrecht, The Netherlands; 4 Netherlands Childhood Cancer Parent Organization VOKK, Schouwstede 2b, 3431 JB Nieuwegein, The Netherlands; 5 Emma Children’s Hospital, Amsterdam UMC, Vrije Universiteit Amsterdam, Paediatric Oncology, Cancer Center Amsterdam, De Boelelaan 1117, 1081 HV Amsterdam, The Netherlands; 6 Princess Máxima Center for Pediatric Oncology, Heidelberglaan 25, 3584 CS Utrecht, The Netherlands; 7 Department Paediatric Oncology, University Medical Center Groningen, Hanzeplein 1, 9713 GZ Groningen, The Netherlands

**Keywords:** Pediatric oncology, Systematic review, Meta-analysis, Hypnotherapy, Procedural pain, Decision-making

## Abstract

**Purpose:**

To develop an evidence-based decision aid for parents of children with cancer and to help guide them in the use of complementary and alternative medicine (CAM) for cancer care.

**Methods:**

This study had a mixed research design. The needs of parents were investigated by survey and focus group. A systematic review and meta-analysis were performed on the effectiveness of CAM using Grading of Recommendations Assessment, Development and Evaluation (GRADE). Clinical experts were interviewed and a decision aid on CAM treatment for pain was developed.

**Results:**

Parents emphasized the importance of reliable information on CAM, focusing primarily on communication and a broad spectrum of complaints related to cancer treatment. The decision aid on CAM for pain included five modalities based on 11 randomized control trials (RCTs): hypnotherapy, mind-body techniques, massage, healing touch, and music therapy. Meta-analysis could be performed on hypnotherapy, which significantly reduced cancer-related procedural pain compared with standard care (MD, − 1.37; 95% CI, − 1.60, − 1.15; *P* < 0.00001) and attention control (MD, − 1.13; 95% CI, − 1.34, − 0.94; *P* < 0.00001), and massage, demonstrating no effect on pain compared with standard care (MD, − 0.77; 95% CI, − 1.82, 0.28; *P* = 0.15). Research evidence and supplementary information from clinical practice and patient were incorporated in a website-based decision aid.

**Conclusions:**

An evidence-based decision aid was developed to support parents of children with cancer in making decisions about CAM for pain management. Next steps will be to expand the website to include additional childhood cancer-related complaints and to evaluate its use in practice.

**Electronic supplementary material:**

The online version of this article (10.1007/s00520-019-05058-8) contains supplementary material, which is available to authorized users.

## Introduction

Complementary and alternative medicine (CAM) can be defined as healthcare approaches that are not typically part of conventional medical care or that may have origins outside of usual Western practice [1]. Although not part of mainstream medicine, an increasing number of children with cancer use CAM along with conventional therapies [2]. Parents choose CAM for treatment and cure of cancer in their children [3], and also as supportive care agent to help minimize side effects of cancer treatment and to improve the general quality of life and well-being [4, 5]. The prevalence of CAM use among children with cancer is high and varies between 6 and 100%, depending on the survey sample and country, and is on average 47.2% in high-income countries [3]. CAM modalities most commonly used are herbs, diets and nutrition, homeopathy, and prayer [4, 6, 7]. Despite the high prevalence of CAM use in children with cancer, it is difficult for parents to find reliable information about it. Existing platforms developed to advise patients on CAM use for cancer, for example the website of the National Cancer Institute [8], are directed towards cancer in adults and do not specifically discuss the suitability of CAM modalities for use in children. Other information available on the internet is of poor quality [9] and frequently includes the statement: “Always consult with your child’s physician before beginning any CAM, because some therapies may interfere with standard treatment.” However, physicians typically do not receive training in CAM and have limited knowledge on the subject and so find themselves unable to discuss any possible (side) effects of CAM [10, 11]. Additionally, it is known that few parents even discuss CAM use with the pediatric oncologist [7, 12, 13]. Authoritative and reliable access to information is thus needed to support parents in their search for information on CAM modalities as potential supportive treatment options for their children with cancer.

This study describes the process and outcome of a project that aimed to develop and implement an evidence-based decision aid on CAM use for pain in order to enable parents of children with cancer to make well-informed decisions concerning CAM. Since children with cancer use CAM most often as supportive care agent [14, 15], the decision aid focused on supportive CAM care for pain.

## Methods

### Project design

**Fig. 1:**
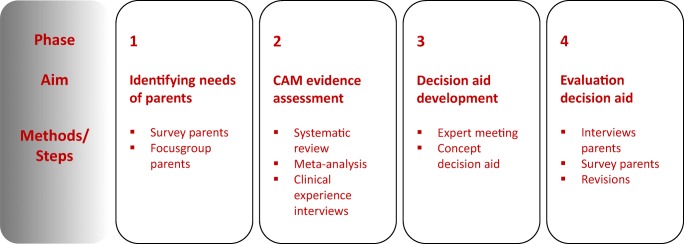
Project overview

The project had four phases of mixed methodology design (see Fig. 1) and was carried out from 2014 to 2017 by a multidisciplinary team of pediatric oncologists, members of the patient organization for parents of children with cancer (VOKK), CAM experts, and researchers. A scientific advisory board consisting of six pediatricians/pediatric oncologists supported the project team.

### Phase 1: needs of parents

The needs of parents with respect to a decision aid on CAM use for their children with cancer were investigated by means of a survey and focus group.

#### Survey

In April–May 2015, a 23-item anonymous questionnaire was distributed by the VOKK to parents of children with cancer via their e-newsletter and Facebook campaign. The questionnaire was modified from previously used questionnaires on self-management instruments [16] and CAM [7, 17]. It was validated through a pilot survey among eight parents and revised accordingly with respect to rewording terms to prevent misinterpretation. The questionnaire is attached as online resource 1. Descriptive statistics were used to analyze the data by means of SPSS 22.0.

#### Focus group

A focus group took place in June 2015 with seven parents of children with cancer that were recruited by the VOKK. The first part of the focus group used the brainwriting technique [18] in response to the following question: What do you need in order to make a good choice about CAM use for your child? Next, the following topics were discussed: possible reasons to use CAM, information and decision-making on CAM use, communication about CAM with physicians/oncologists, and any additional requirements for a decision aid on CAM. The focus group lasted 2 h and was recorded, and field notes were taken. A directive content analysis [19] was performed to get a better understanding of the parents’ needs regarding a decision aid on CAM.

### Phase 2: evidence on CAM

Evidence for CAM was investigated by means of a systematic review and meta-analyses. It was limited to pain management in children as it was not feasible within the project grant and time to perform additional systematic reviews on other cancer-related complaints.

#### Systematic review and meta-analysis

The review was planned and conducted in accordance with the Preferred Reporting Items for Systematic Reviews and Meta-Analyses (PRISMA) guidelines [20]. The protocol was not registered in a database. The PICOS (Patient-Intervention-Comparison-Outcome-Study) design for the systematic review was defined as follows: Effects (reduction) on experienced pain during cancer treatment and post-treatment and occurrence of adverse events (outcome) of CAM (intervention) in comparison with no treatment, standard treatment, and placebo control or active control group (comparison) in children with cancer (patient) reported in prospective controlled studies (study). Between December 2015 and March 2016, the PubMed, EMBASE, Cochrane, and CINAHL were searched. Search terms and strings are attached as online resource 2. Inclusion criteria were peer-reviewed prospective controlled studies in children (0–18 years) with any type of cancer undergoing a CAM intervention (as defined by the National Center for Complementary and Alternative Medicine [21]) published in English or Dutch language. The primary outcome was pain (any pain-related outcome), and the secondary outcome was reported CAM-related adverse events. Two researchers performed the selection of studies, data extraction, and risk of bias assessments. Any instances of disagreement were resolved by consultation with a third researcher. The risk of bias assessment was done according to the Grading of Recommendations Assessment, Development and Evaluation (GRADE) handbook [22]. The GRADE approach was used to facilitate the overall quality of each outcome and to assess risk of bias across studies [23]. Meta-analysis was performed in cases where the results of two or more randomized clinical trials (RCTs) per CAM modality could be pooled using RevMan 5.3 (Cochrane collaboration) and GRADEpro (version 3.6) for different scores related to pain across interventions. *I*^2^ was used to test for heterogeneity in judging consistency of evidence.

#### Clinical experience

Six pediatric oncologists working at integrative pediatric oncology clinics in Canada, the USA, Germany, and Italy were interviewed to collect information related to the use and safety of CAM in the treatment of pain in children with cancer. These experts were identified at the International Congress on Integrative Medicine and Health (Las Vegas, 2016). The interview guide consisted of seven questions (see online resource 3). Interviews were conducted face-to-face or via Skype, were recorded, and varied in length from 30 to 60 min. The content was analyzed and categorized as follows: CAM modalities, effects, treatment phase, type of pain and patient, complaints, and side effects.

### Phase 3: development decision aid

The objective of phase 3 was to combine information on research evidence and clinical expertise in order to inform recommendations and decision-making regarding CAM use for pain management in children with cancer. A 3-h expert meeting was organized in June 2016 consisting of a balanced mix of pediatric oncologists, pediatricians, pediatric nurses, parents, CAM experts, researchers, and members of the patient organization. The meeting included 14 participants in total. Preceding the expert meeting, all participants read the outcome and underlying documentation of phase 2 and were informed about the procedure and steps in the meeting, which followed the seven-step approach of GRADE [22]. Other CAM-related contextual factors taken into consideration included whether the CAM treatment was safe, feasible to implement, could be taught and practiced by the children or the parents independently, was practiced individually or as a group, and whether it was a facilitator for conventional treatment. Based on the recommendations and decisions that were reached in the expert meeting and on the results from the earlier phases, three text writers (representing the patient, CAM, and scientific perspective) wrote the content for the decision aid. The concept structure of the decision aid was developed by members of the project team in parallel with technical support from KSMT design (The Hague, the Netherlands).

### Phase 4: evaluation of decision aid

The content and structure of the decision aid were evaluated through interviews and a short questionnaire (see online resource 4) administered to the VOKK, three pediatric oncologists, and 13 parents. Evaluation was based on the look and feel, structure, ease of comprehension, usability, navigation, usefulness in decision-making, and interface. Analysis of feedback was performed by one researcher and the content was adapted accordingly.

## Results

### Phase 1: needs of parents

In order to develop a practical and useful decision aid on CAM use that is likely to be adopted by families affected by cancer, the project started with investigation of the needs of the parents.

#### Survey

Among those surveyed, 70 parents responded. Mothers made up the majority of respondents (93%). Among all respondents, most had a child with leukemia (55%) or a brain tumor (17%). Among the cancer-affected children of respondents, 31% were currently in treatment and 38% had finished treatment more than 1 year ago. More than half (56%) reported CAM use for their child. Among those who reported CAM, the use of food supplements/vitamins (32%), massage (22%), and homeopathy (22%) were most mentioned. One-third of those using CAM for their child had searched for information about the CAM treatment before use. Sources included the Internet (37%), family/friends (37%), and CAM practitioners (26%). Eighty-eight percent of respondents thought it was important to receive/find good quality information on CAM. Most respondents (79%) had a need for information on CAM following their child’s cancer diagnosis, including 38% just after diagnosis, 45% during the first year after cancer treatment, 34% more than a year following cancer treatment, and 14% in the palliative phase. Parents sought information on CAM for treatment of fatigue (62%), anxiety (47%), pain (46%), weakened immune system (44%), sleeping problems (42%), nausea/vomiting (35%), low mood (29%), decreased appetite (27%), intestinal problems (22%), concentration problems (16%), and weight loss (11%). Parents mentioned a need for a decision aid to support their discussions of CAM treatments with their physician (60%), to find reliable CAM practitioners (59%), for education regarding possible CAM use (45%), for support in asking questions of CAM experts (43%), and as a resource for reading about other parents’ experiences with CAM (40%). Preferences for the form of the decision aid were website (62%), app (23%), or informational brochure/leaflet (12%). Preference of most respondents was that the decision aid be accessible via the treating pediatric oncologist (88%) or VOKK (83%).

#### Focus group

Direct content analysis was performed on five themes: (1) Search strategies: Parents usually start searching for CAM options after their child has been receiving treatment for some time as closer to time of diagnosis they have too much information to process. Parents report searching the Internet for CAM modalities effective for specific complaints or cancer types, for finding reliable CAM practitioners, and for information on whether particular CAM modalities can be combined with cancer treatment. (2) Decision-making: Decisions on the use of CAM are mostly based on the positive experiences of family/friends, on parent’s personal experiences with CAM, or on the availability of evidence for a particular CAM modality. (3) Format of the decision aid: A website with a search function for complaint, type of cancer, and CAM modality, along with a chat function allowing interaction with other parents and a CAM expert who can answer CAM-related questions. (4) Function of the decision aid: To allow the parents make their own decisions with regard to CAM, and also as a tool to discuss the outcome on CAM with the pediatric oncologist. (5) Content of the decision aid: Description of positive effects of particular CAM modalities, evidence for their use, potential side effects, and possible interference with cancer treatment.

### Phase 2: evidence on CAM

Based on the results of phase 1, the project team needed to make a decision on the focus of the decision aid. Since there are more than 1800 CAM modalities [24] and performing systematic reviews to collect evidence-based information on all these modalities is time-consuming, it was not feasible to develop a decision aid on all childhood cancer-related complaints for which parents in phase 1 wanted information about. It was decided to focus on CAM use for pain during cancer treatment and post-treatment because (1) pain was in the top three complaints that parents wanted information about in phase 1 and (2) the Dutch Childhood Oncology Group (DCOG; www.skion.nl) was developing clinical guidelines for treatment of pain, so a joint venture was planned.

#### Systematic review on CAM for pain

**Fig. 2:**
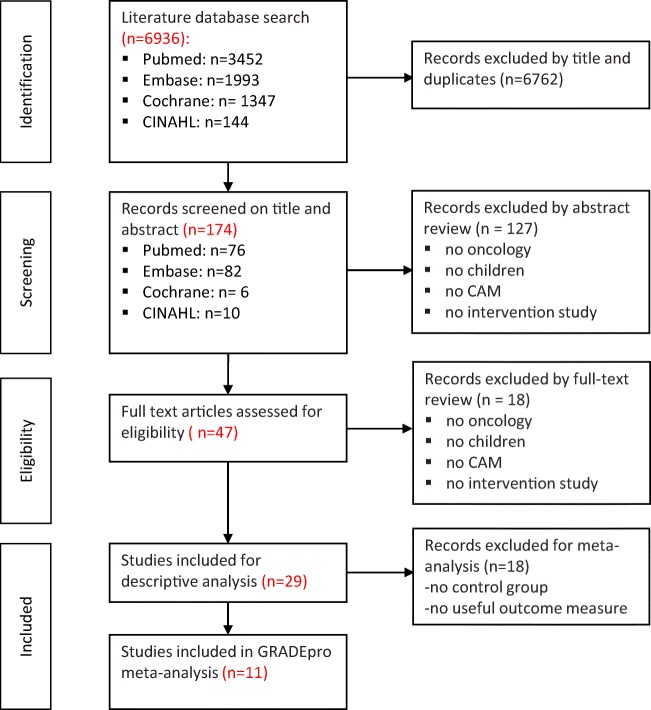
Flow diagram of study selection and identification

Figure 2 shows the study selection process. A total of 6936 records were identified from searches, of which11 RCTs met the criteria for GRADE analysis [25–35]. An additional 18 articles served as input for phase 3 (see online resource 5). The characteristics of the included RCTs are summarized in Table 1. RCTs were found for five CAM modalities: hypnotherapy (*N* = 5), massage (*N* = 3), healing touch (*N* = 1), music therapy (*N* = 1), and mind-body intervention (*N* = 1). Ten out of 11 studies investigated the changes in pain related to procedures such as venapuncture, lumbar puncture, or bone marrow aspiration. The 11 studies included children with all types of cancer. The quality of studies was moderate to high for the RCTs on hypnotherapy and low to moderate for the other CAM modalities (Table 1). Since the studies differed with respect to pain scales, control groups, and number of RCTs per CAM modality, pooled effects on the primary outcome could only be analyzed for hypnotherapy and massage (Table 2). There was high-quality evidence that hypnotherapy significantly reduces cancer-related procedural pain in children; the pooled effect was statistically significant (MD, − 1.37; 95% CI, − 1.60, − 1.15; *P* < 0.00001) compared with standard care [27–29], and statistically significant (MD, − 1.13; 95% CI, − 1.34, − 0.94; *P* < 0.00001) compared with an attention-control group [26–29] (Table 2). Both analyses showed significant heterogeneities (*P* < 0.001), with *I*^2^ values of 87% in comparison with standard care and 86% in comparison with attention-control. One high-quality study on hypnotherapy by Smith et al. [34] was not included in the meta-analysis because it used a different VAS scale and outcome data were only reported for children with high hypnotizability and children with low hypnotizability. There was low-quality evidence for no effect of massage (Swedish/acupressure and effleurage/petrissage techniques) on cancer-related procedural pain compared with standard care; the pooled effect of two studies was not significantly different (MD, − 0.77; 95% CI, − 1.82, 0.28; *P* = 0.15, heterogeneity *I*^2^ = 0%, *P* = 0.80) (Table 2). No evidence was found that the five CAM modalities were unsafe to treat pain in children with cancer.

**Table 1:** Literature review table—included studies

Study	Design	Sample	Intervention(s)	Findings	Findings short*	Quality of evidence	Quality level
Hypnotherapy							
Katz 1987 [22]	RCT, evaluating **hypnotherapy** versus attention control for pain, anxiety, and distress associated with BMAs	Children with acute lymphoblastic leukemia (6–11 years) undergoing repeated BMA who experience significant anxiety, fear, and/or pain during BMA (*n* = 36)	**Hypnotherapy**: hypnotic induction, active imagery, individually tailored, deep muscle relaxation, and suggestions. Ending with a post-hypnotic suggestion Attention control: non-directed play sessions designed to control for the amount of time and attention	- Improvement was reported in self-reported pain (0–100 VAS) and distress over baseline with both interventions, with no differences between them. - No significant main effects were found in PBRS scores. - Girls exhibited more distress behavior than boys on three of four dependent measures used. - Results are discussed in terms of potential individual differences in responding to stress and intervention that warrant further research	*Hypnosis* vs *attention control* = no differences for pain and distress *Post-treatment* vs *baseline* + pain and distress (for hypnosis and control)	RCT, sufficient sample size, randomization process not entirely described, blinding of independent observers, nurses and observers, good inter-rater reliability. No selective reporting, adequate analysis, study completed as planned, no missing data	Moderate
Smith 1996 [30]	RCT, cross-over, repeated measures single group study evaluating **hypnosis** versus distraction for pain, anxiety, and distress associated with venipuncture or infusaport access	Children (3–8 years) with hematology and oncology diagnoses undergoing repeated venipuncture or infusaport access (*n* = 27)	**Hypnosis:** favorite place hypnotic induction. Both parents and children were taught the exercises. Attention control/distraction: activating the pop-up toy, noting interesting aspects of the toy	- Only children with high hypnotizability had reduced child self-reported pain (1–5 Global Rating Scale) and anxiety (1–5 Likert scale), parent-rated pain (1–5 Likert scale), and observer anxiety and distress from hypnosis intervention (OSBD-R scale) -Children with low hypnotizability in the distraction condition had significantly lower observer-rated anxiety only -Practical: parents and children were both trained in hypnosis exercises. Parents were very positive and exercises were easy to learn and practise.	*Hypnosis* vs *control* ++ for self-reported pain ++ for parented reported pain ++ for distress All only for children with high hypnotizability	RCT, cross-over design. Observers, trainers, and parents were told that both interventions were equally effective, observers were blind to high and low hypnotizability level of children, both self-reported measurements and observer measures. Adherence to the exercises at home was monitored and no significant differences in compliance were observed between the groups. Sufficient sample size, no selective reporting, adequate analysis, study generally completed as planned, some missing data due to death of participants	High
Liossi 2003 [23]	RCT, evaluating **direct hypnosis** and **indirect hypnosis** versus attention control and standard care for pain and distress associated with LPs	Children and adolescents (6–16 years) with leukemia or non-Hodgkin lymphoma undergoing repeated LPs (*n* = 80)	**Indirect hypnosis**: Using metaphors, imagination using various senses, develop cues to experience immediate relaxation, and ways to adapt to discomfort. Ending with a post-hypnotic suggestion. Directed by therapist and then self. **Direct hypnosis:** “Analgesic” suggestions. Directed by therapist and then self. Attention control: including elements such as development of rapport, non-medical play, and no medical verbal interactions Equivalent time was spend with the therapist as in hypnotherapy. Standard care: no contact with the therapist, medical care for pain with LP provided by the hospital staff.	-Direct and indirect hypnosis groups were equally effective and reported less pain and anxiety (both 0–5 Wong-Baker Faces scale) as compared with attention control or standard care groups. -Higher levels of child hypnotizability associated with increased treatment benefit. -Treatment benefit lessened with self -hypnosis as compared with therapist-directed	*Hypnosis* vs *attention control or standard care* + for pain and distress (indirect and direct hypnotherapy) *Indirect* vs *direct hypnosis* = for pain and distress	RCT, sufficient sample size, independent observers, doctors, and behavioral observers were blinded, blinding was measured, observers could only guess which children were in the direct hypnosis group (intervention 1), they could not distinguish between the other intervention groups and control group, no selective reporting, appropriate analysis, study completed as planned, no missing data.	High
Liossi 2006 [24]	RCT evaluating **hypnosis** versus attention control or standard care for pain and distress associated with LPs	Children and adolescents (6–16 years old) with leukemia or non-Hodgkin lymphoma undergoing repeated LPs (*n* = 45)	**Hypnotherapy**: Standard care + “Analgesic” suggestions, ending with a post-hypnotic suggestion. Directed by therapist and then self. Attention control: Standard care + including elements such as development of rapport, non-medical play, and no medical verbal interactions Equivalent time was spend with the therapist as in hypnotherapy Standard care: EMLA/analgesic cream. Medical care for pain with LP provided by the hospital staff	- Group receiving hypnosis, in addition to local anesthetic (EMLA), reported less pain and anxiety (both 0–5 Wong-Baker Faces scale), and less observed behavioral distress as compared with other groups. -Treatment superiority was maintained when switched to self -hypnosis following therapist-directed hypnosis. -Higher levels of child hypnotizability associated with increased treatment benefit	*Hypnosis* vs *attention control or standard care* ++ for self-reported pain and distress	RCT, sufficient sample size. Independent observers, doctors and behavioral observers were blinded. Blinding was measured, observers could not guess in which groups the children were allocated. Inter-rater reliability was tested and found to be good. No selective reporting, appropriate analysis, study completed as planned, no missing data	High
Liossi 2009 [25]	RCT evaluating **self-hypnosis** versus attention control or standard care for pain and distress associated with venipuncture	Children and adolescents (7–16 years) with cancer undergoing venipuncture (n = 45)	**Self-hypnosis**: Standard care + “Analgesic” suggestions, ending with a post-hypnotic suggestion. Following that, children were taught self-hypnosis. Attention control: standard care + including elements such as development of rapport and no medical verbal interactions. Equivalent time was spend with the therapist as in hypnotherapy Standard care: EMLA/analgesic cream. Medical care for pain with LP provided by the hospital staff	-Self-hypnosis + local anesthetic (EMLA) reported less anticipatory and experienced anxiety, pain (self-report 100 mm VAS) and observed behavioral distress as compared with other groups. -Parents experienced less anxiety in the hypnosis group	*Self-hypnosis* vs *attention control or standard care* ++ for self-reported pain and distress + anxiety parents	RCT, sufficient sample size. Independent observers, doctors, and behavioral observers were blinded, blindness was measured, observers could not guess in which groups the children were allocated. Inter-rater reliability was tested and found to be good No selective reporting, appropriate analysis, study completed as planned, no missing data.	High
Mind-body (including imagery, meditation, breathing techniques)							
Pourmovahed 2013 [29]	RCT evaluating **regular breathing versus** standard care for pain associated with intrathecal injections	Children and adolescents (6–15 years) with leukemia undergoing a first intrathecal injection (*n* = 100)	**Hey-Hu breathing technique**: the child first takes a deep breath and exhales while whispering ‘hey’, then inhales deeply again and exhales whispering “hu” Standard care: current standard medical practice	- Children in the “Hey-Hu” breathing group reported significantly less pain (0–5 Wong-Baker Faces scale) than the control group, particularly among children aged above 10 years. -There was no significant difference between the two sexes. -Nurses could help children learn the method of ‘Hey-Hu’ breathing and implement it in hospitalized children who undergo painful procedures.	*Hey-Hu breathing* vs *standard care* + for pain ++ for pain in children > 10 years	RCT, sufficient sample size, sampling using random allocation software, some blinding **(s**emi-blind, the performer of the procedure was aware of the aim of the study), no selective reporting, appropriate analysis, study completed as planned, some missing data.	Moderate
Massage							
Phipps 2005 [28]	RCT, unbalanced pilot, evaluating **professional massage** and **parent massage** versus standard care for pain and distress experienced undergoing hematopoietic stem cell transplantation (HSCT)	Children (all ages) scheduled to undergo HSCT (*n* = 50)	**Professional massage**: therapeutic massage delivered by licensed massage therapists three times per week for the 4-week period from admission for HSCT through 3 weeks post-transplantation. **Parent massage**: parents learned basic massage techniques to use with the child. The routines taught to the parents were essentially the same as those provided in the therapist massage arm. Parents were asked to begin giving their child massage at least three times per week. - Standard care: usual care	-No significant differences were observed between the two massage interventions on distress and pain scores (self-report 100-mm VAS). -No significant differences between either massage group and standard care for pain and distress, although there were descriptive trends suggestive of benefit, some of which approached significance. Larger differences emerged on the outcomes of days in hospital and days to engraftment, pointing to the potential cost-benefits of a massage intervention in this setting. -Regarding narcotic usage, there were no significant differences between groups, but descriptively there was a trend for those in the massage arms to use less medication.	*Professional* vs *parent massage* = no differences for pain and distress between massage groups *Massage* vs *standard care:* = for pain, distress and narcotic medication use (for professional and parental massage)	RCT, insufficient sample size (underpowered, though the sample was representative of the population of patients who underwent transplantation), allocation to treatment arms was not equal but was designed so that participants were twice as likely to enter either intervention arm than the control arm, lack of blinding, no selective reporting, appropriate analysis, not described if study completed as planned, some missing data reported	Low
Mehling 2012 [26]	RCT, non-blinded pilot, feasibility study, evaluating a **combined massage-acupressure intervention** versus standard care, for decreasing treatment-related symptoms such as nausea, vomiting and pain associated with hematopoietic cell transplant	Children (5–18 years) undergoing hematopoietic cell transplant at an academic medical center (*n* = 23)	**Combined massage-acupressure intervention**: practitioner-provided, combined Swedish and acupressure massage three times a week throughout hospitalization. Parents were trained to provide additional acupressure as needed. - Standard care: usual care	-There was no statistically significant difference or change in pain (BASES subscale self-report) between the two groups -Intervention group versus control showed fewer days of mucositis, lower overall symptom burden, feeling less tired and run-down, having fewer moderate/severe symptoms of pain, nausea, and fatigue	*Massage* vs *control* = for pain	RCT, insufficient sample size (small feasibility study, aim to report standardized effect sizes that allow for sample-size calculations for future studies), no blinding, no selective reporting, appropriate analysis, study completed as planned, no missing data	Low
Celebioglu 2015 [21]	Controlled pretest/posttest quasi-experimental study, investigating the effect of **massage therapy** versus standard care, on pain and anxiety arising from intrathecal therapy or BMA	Children (4–15 years) with primary diagnosis of cancer (*n* = 25)	**Massage therapy**: one massage session from a licensed massage therapist. Massage techniques were a combination of effleurage and petrissage to the shoulders, neck, face, arms, lower back and waist. Standard care: standard treatment offered to patients undergoing IT or BMA.	-No difference between groups for pain (0–10 VAS self-report or by mother) or anxiety -It was determined that pain and anxiety levels in the massage group decreased significantly post-treatment versus baseline	*Massage* vs *control* = for pain and anxiety *Post-treatment* vs *baseline:* + for pain and anxiety (massage group)	Pretest/posttest quasi-experimental study with the control group, small sample size, non-probability convenience sampling, children were divided between the groups according to admission date, no blinding, no selective reporting, inappropriate analysis, study completed as planned, no missing data	Low
Healing touch							
Wong 2013 [31]	RCT, evaluating **healing touch** versus attention control on feasibility in pediatric oncology	Children (3–18 years) diagnosed with childhood malignancy, receiving chemotherapy and/or radiation therapy (*n* = 9)	**Healing touch**: by certificated practitioner, standardized techniques. Attention control: reading or age-appropriate play activity for the same time as the intervention group	-There were statistically significant differences in pain scores (children and parents on 1–10 Wong-Baker Faces scale) and distress scores (parents) between the healing touch group and the control group. - Among the healing touch group, all scores (pain, distress, and fatigue) decreased significantly after the intervention. Scores among the control group did not show a statistically significant decrease. - The study demonstrates the feasibility of using energy therapy in the pediatric oncology patient population.	*Healing touch* vs *control* ++ for self-reported pain + for pain reported by parents = for pain reported by staff = for self-reported distress + for distress reported by parents = for distress reported by staff	RCT, insufficient sample size (recruitment rate 60%), the participants in the intervention group received approximately 6.5 times more treatments than the control group, which may bias results. High heterogeneity of groups (age, diagnose, and treatment protocols), no blinding. No selective reporting. Inappropriate analysis, study not entirely completed as planned (2 drop-outs, because of prolonged hospitalizations and complicated treatments and 1 participant died while in the study because of disease progression), some missing data	Low
Music therapy							
Nguyen 2010 [27]	RCT, evaluating **music** vs control for pain and distress associated with LPs	Children (7–12 years) with leukemia undergoing LPs (*n* = 40)	**Music group** (earphones with music): Children choose their own music to be played into earphones from an iPod, 10 min before the LP procedure started. Control group (earphones without music): same procedure as music group, only without music	- As compared with the control group, children in the music group had significant reduction in self-reported pain (0–10 Numeric Rating Scale during and after procedure) and anxiety (before and after the procedure) -Significant reductions in heart rate and respiratory (during and after procedure) in music group; blood pressure and oxygen saturation did not differ between groups - The findings from the interviews confirmed the quantity results through descriptions of a positive experience by the children, including less pain and fear.	*Music* vs *control* ++ for self-reported pain during and after the lumbar puncture. ++ for heart- and respiratory rates during and after the lumbar puncture. = for blood pressure and O_2_ saturation	RCT, sufficient sample size, lack of blinding (all the children were given identical pre-procedural information, randomization was carried out using opaque envelopes, the researcher and the physician did not know to which group the patient belonged), no selective reporting, correct analysis, study completed as planned, no missing data	High

*RCT*, randomized controlled trial; *LP*, lumbar puncture; *BMA*, bone marrow aspiration; *IV*, intravenous; *CBT*, cognitive-behavior therapy; *GA*, general anesthesia; *IM*, intramuscular injection

*: + or − → *P* < 0.05

++ → *P* < 0.001

= → no significant difference

**Table 2:** GRADE evidence profile

Quality assessment							No. of patients		Effect		Quality
Included studies	Study design	Risk of bias	Inconsistency	Indirectness	Imprecision	Other considerations	Intervention	Control	Absolute (95% CI)	*P* value	
Self-reported pain on VAS scale (0–5)							Hypnotherapy	Standard care			
Liossi 2003, Liossi 2006, Liossi 2009	Randomized trials	Not serious	Not serious	Not serious	Not serious	None	50	50	MD − 1.37 lower* (− 1.6 to − 1.15)	< 0.00001	⨁⨁⨁⨁HIGH
Self-reported pain on VAS scale (0–5)							Hypnotherapy	Attention control			
Liossi 2003, Liossi 2006, Liossi 2009, Katz 1987	Randomized trials	not serious	not serious	not serious	not serious	none	67	69	MD − 1.13 lower* (− 1.34 to − 0.93)	<0.00001	⨁⨁⨁⨁HIGH
Visual analog scale (VAS), range 0–10, self-reported							Massage	Standard care			
Celebioglu 2015, Mehling 2012	Randomized trials	Very serious	Not serious	Not serious	Not serious	None	28	20	MD − 0.77 lower* (− 1.82 to 0.28**)	0.15	⨁⨁◯◯LOW

*CI*, confidence interval; *MD*, mean difference. *A negative effect value favors the CAM intervention group, a positive effect value favors the control group. **In case the CI includes the null-value, it indicates there is no significant difference between the groups

Since it is more challenging to conduct trials in children than in adults [36], especially with regard to cancer, the number of pediatric patients in studies is still limited [37]. Therefore, in order to collect more information on the appropriate use of specific CAM modalities for pain management in children with cancer, it was decided to investigate the clinical experiences of pediatric oncologists with expertise in CAM. Experts had positive clinical experience with all five CAM modalities that were found in the systematic review (see online resource 6).

### Phase 3: development decision aid

Data of the systematic review and the clinical experience from pediatric oncologist, as obtained in phase 2, formed the main input for the development of the decision aid in phase 3. A crucial step in phase 3 was to reach consensus among stakeholders with respect to recommendations on CAM use. Five CAM modalities (hypnotherapy, mind-body techniques, massage, healing touch, and music therapy) were included in the decision aid based on the results from the systematic review. The clinical expert opinions of pediatric oncologists in Integrative Oncology as well as patients’ needs and preferences were considered important supplementary sources of information for these five CAM modalities. Recommendations were given for each CAM modality (see Table 3). The project team processed the information from the phase 2 literature analysis and expert meeting into a concept structure for a website-based decision aid, and the content was written by three text writers.

**Table 3:** Recommendations on CAM use for pain management in children with cancer

CAM modality	Evidence	Recommendation	Supplementary information
Hypnotherapy	There is high-quality evidence for a positive effect. No evidence for side effects.	Hypnotherapy is recommended for the prevention and/or reduction of procedural pain.	For children of 6 years of age and older; requires concentration and imagination; for procedural and chronic pain, little to no side effects; when the child experiences dizziness, exercises can be performed when laying down; can be guided by a hypnotherapist, a CD or app with exercises or simple exercises can be taught to the parent as to guide their child.
Mind-body techniques	There is low-quality evidence for an inconsistent effect. No evidence for side effects	Mind-body techniques may be considered for the prevention and/or reduction of procedural pain.	For children of 3–4 years of age and older; mind-body techniques such as breathing and relaxation techniques, meditation, and guided imagery; for procedural pain and pain related to stress and anxiety; supportive care option in combination with physiotherapy; no reported side effects; can be guided by trained nurse or psychologist, a CD or app with exercises or simple exercises can be taught to the parent as to guide their child.
Massage	There is low-quality evidence for no effect. No evidence for side effects	Massage is not recommended for the prevention and/or reduction of pain but may be considered to support general well-being.	For all ages; in each treatment phase; often young children prefer to receive massage from their parents; no reported side effects; massage is contraindicated for open wounds and skin lesions and low platelet counts; can be provided by physiotherapist, nurse, or simple exercises can be taught to the parent.
Healing touch	There is low-quality evidence for a positive effect.	Healing touch may be considered for the prevention and/or reduction of pain.	For all ages; in each treatment phase; specifically suitable for children that do not like to be touched; no reported side effects; may not align with parents believes or religion; can be provided by trained nurse, therapist or parent can follow a course.
Music therapy	There is low-quality evidence for a positive effect.	Music therapy may be considered for the prevention and/or reduction of procedural pain.	For all ages; in each treatment phase; no reported side effects; can be provided by music therapist, a CD or app.

### Phase 4: evaluation and implementation of decision aid

The content and structure of the decision aid as developed in phase 3 was evaluated by the VOKK, parents, and pediatric oncologists. Feedback included comments on the inclusion of too much scientific terminology that some information was too general in nature, to improve clarity with regard to which CAM modalities are supported by evidence and with regard to the information on which the content was based. According to the preference of parents (see “Phase 1: needs of parents” under the “Results” section), the decision aid was linked to the VOKK, the Dutch organization for parents of children with cancer: http://www.complementairezorg-vokk.nl/.

**Fig. 3:**
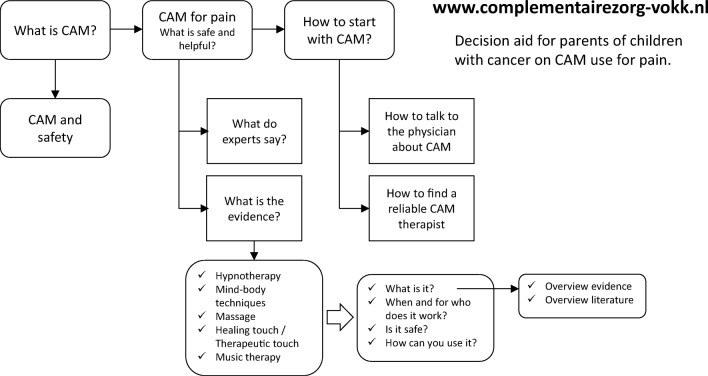
Visual overview structure decision aid

The overall structure of the decision aid is depicted in Fig. 3. To facilitate its use, the VOKK announced the launch of the decision aid via the newsletter, website, and Facebook page and via an article in their printed news magazine. A “business” card of the decision aid was developed for the Princess Máxima Center for pediatric oncology, the recently established high-complex care center for childhood cancer in the Netherlands [38], to make parents aware of the existence of the decision aid. Awareness among healthcare professionals was created via DCOG, and the decision aid was introduced in education programs at the Princess Máxima Center and at the University Medical Center in Utrecht. The Louis Bolk Institute assumed ownership and responsibility for maintenance and updating of the decision aid.

## Discussion

To the best of our knowledge, this is the first study that reports on the development of an evidence-based decision aid for supportive CAM care on pain in childhood cancer. The decision aid was primarily developed as a website for parents, but is considered more than an information website. A decision aid is defined as an evidence-based tool designed to help patients make specific and deliberate choices about available healthcare options [39]. In order to facilitate patient-centered decision-making, the totality of evidence in a decision aid should comprise four pillars: research evidence, practice evidence, patient evidence, and contextual factors [40]. First, the decision aid provides evidence-based information. In addition to high-quality research evidence, it incorporates information from practice (clinical experience), contextual factors (feasibility of implementation), and patient preferences. Second, it is patient-centered and facilitates decision-making by parents of children with cancer, for example helping them to decide whether or not to use CAM as supportive care for their child. Third, it supports parents to recognize possible benefits and harm related to their decision about CAM use for their child. Finally, the decision aid is designed to be helpful for parents in discussions of CAM with healthcare professionals. Although few parents currently discuss CAM use with the treating pediatric oncologist [7, 12, 13], the majority want to receive information and discuss it with them [7]. The use of the decision aid as a tool has the potential to improve communication on CAM between parents and healthcare providers.

One of the major limitations of the current decision aid is that it is limited to decision-making on CAM modalities for pain relief. The potential effectiveness of the five CAM modalities included in the decision aid, or of other CAM modalities in addressing other complaints than pain, was not investigated. Recently, a RCT in children undergoing hematopoietic stem cell transplants reported that although music therapy did not significantly reduce pain compared with control, it significantly enhanced health-related quality of life [41]. Another major limitation of this study is that it did not evaluate whether the current decision aid actually improved parents’ knowledge regarding CAM treatment options, or whether it reduced the decisional conflict stemming from feeling uninformed and unclear about treatment options. Future studies are planned to evaluate the effectiveness of the decision aid for parents in practice and, additionally, to assess its use in communication between parents and healthcare professionals. In the present study, parents wanted information on CAM for a broad spectrum of symptoms related to childhood cancer treatment, i.e., fatigue, anxiety, and pain. This is in line with a previous study, demonstrating that parents perceive high symptom burden in their children with cancer, pain being the most problematic area [42]. Next steps recommended concern expanding the decision aid on CAM for other pediatric cancer-related complaints, as to provide further evidence on how CAM modalities may contribute to reduce the symptom burden in children with cancer. In addition, further investigations are warranted as to disclose potential harmful interactions between CAM remedies and cancer drugs.

A strength of the present study is that the decision aid was developed based on the true needs of parents and was developed by a multidisciplinary team who consider CAM worthy of serious evaluation, so that the perspectives and contextual factors of stakeholders in childhood cancer were taken into account.

In conclusion, an evidence-based decision aid was developed to support parents of children with cancer in making decisions on possible CAM treatment for pain management. Next steps will be to expand the website with evidence for CAM on other childhood cancer-related complaints and to evaluate its use in practice.

## Electronic supplementary material

ESM 1 (DOCX 30.8 kb)

ESM 2 (DOCX 33 kb)

ESM 3 (DOCX 24.7 kb)

ESM 4 (DOCX 23.6 kb)

ESM 5 (DOCX 45 kb)

ESM 6 (DOCX 28 kb)
