# Supplementary material for: Development of an evidence-based decision aid on complementary and alternative medicine (CAM) and pain for parents of children with cancer
Source: Support Care Cancer. 2019 Sep 6;28(5):2415–29. doi: 10.1007/s00520-019-05058-8 (PMC7083801; doi:10.1007/s00520-019-05058-8)
Supplement: Supplementary file 1 — (DOCX 30.8 kb) [file 520_2019_5058_MOESM1_ESM.docx]

**Online resource 1: Survey questionnaire**Supportive Care in Cancer
Development of an Evidence-Based Decision Aid on Complementary and Alternative Medicine (CAM) for Parents of Children with Cancer.
Miek C. Jong, Inge Boers, Herman van Wietmarschen, Martine Busch, Marianne C. Naafs, Gert-Jan Kaspers, Wim J.E.Tissing.
Dr. Miek C. Jong, Mid Sweden University, Department of Health Sciences, Holmgatan 10, 851 70 Sundsvall, Sweden, email: [miek.jong@miun.se](mailto:miek.jong@miun.se)

**Questionnaire to assess needs of parents of children with cancer**

*What is the need of parents of children with cancer for information about complementary care?*

This questionnaire is developed for parents of children with cancer. With this questionnaire we want to gain insight in whether and how parents want to receive information about complementary care for their child.

*What is complementary care?*

Complementary care literally means ‘additional care’. This doesn’t replace usual care, but is a possible addition to it.

Many parents choose for complementary care for their child to reduce side effects of usual treatment (chemotherapy, surgery and/or radiation). Or because of the fact that their child feels more relaxed because of it. The most commonly used forms of complementary care for children are homeopathy, dietary supplements and massage.

The question here is in which way you as a parent are able to find the most reliable information about complementary care. Reliably information allows making choices that are most fitting for you and your child.

We first ask a number of general questions regarding you, your child and the condition of your child:

1. Gender parent/care taker:

*(It concerns the parent/care taker that is completing this questionnaire)*

- Male
- Female

1. Age parent/care taker:

*(It concerns the parent/care taker that is completing this questionnaire)*

- <20
- 20-25
- 26-30
- 31-35
- 36-40
- 41-45
- 46-50
- 51-55
- >55

1. Highest level of education of parent/care taker:

*(It concerns the parent/care taker that is completing this questionnaire)*

- Primary school
- VMBO / MAVO/ LBO
- MBO
- HAVO / VWO
- HBO
- University

1. Gender child:

- Boy
- Girl

1. Age of child at diagnosis: …….. year
2. Which diagnosis did or does your child have?

- Leukaemia
- Brain tumour
- Lymphoma
- Neuroblastoma
- Kidney tumour
- Soft tissue tumour
- Retinoblastoma
- Bone tumour
- Germ cell tumour
- Liver tumour
- Fanconi anaemia
- Other, eg.:…………..

1. How long ago was the diagnosis?

- Less than 1 month
- More than 1 but less than 3 months
- More than 3 but less than 6 months
- More than 6 but less than 12 months
- More than 1 but less than 3 years
- More than 3 but less than 5 years
- More than 5 years

1. What is the situation now?

- Diagnosis is recent, treatment has not been started
- My child is under treatment (eg. chemotherapy, surgery and/or radiation)
- Treatment was completed less than 1 year ago
- Treatment was completed more than 1 year ago
- My child will not recover and undergoes palliative care
- My child died

The following questions concern your experience with complementary care

1. Did your child make use of one of the following types of complementary care? > question 10

*(More than one answer allowed)*

- Homeopathy
- Dietary supplements / vitamins
- Massage
- Reiki
- Visualisation exercises / relaxation exercises
- Yoga
- Tai Chi
- Mindfulness / meditation
- Phototherapy
- Chiropractic / osteopathic / haptonomy
- Aromatherapy
- Acupuncture / Chinese medicine
- Hypnotherapy
- Naturopathy
- Diet / dietary advise
- Anthroposophy
- Therapeutic touch / healing touch
- Prayer
- Foot reflex therapy
- Ayurveda
- Other, eg.:…………..
- No, my child did not use complementary care

1. Did you find or receive information about complementary care for your child?

- Yes
- No > question 13

1. How did you get this information? > question 11

*(More than one answer allowed)*

- From the hospital
- Internet
- From other parents
- From friends
- From books/magazines/newspapers
- From a CM practitioner
- From my own experience with complementary care
- Other, eg.:………………….

1. Do you think there was enough sufficient quality information to make a good choice? > question 13

- Yes, the information was sufficient, and I chose for complementary care
- Yes, the information was sufficient, but I didn’t choose for complementary care
- No, the information was not sufficient, and I didn’t choose for complementary care
- No, the information was not sufficient, but I did choose for complementary care
- No opinion / I don’t know

The following questions concern your need for information about complementary care for your child.

1. How important is it for you to receive information about complementary care for your child?

- Very important > question 14
- Important > question 14
- Unimportant > alternative end text
- Very unimportant > alternative end text
- No opinion / I don’t know > question 14

1. In which phase of the treatment did you have a need or do you think to have a need for reliable information about complementary care?

*(More than one answer allowed)*

- Directly after the diagnosis
- During treatment (chemotherapy, surgery and/or radiation)
- During the first year after treatment
- During a later stage after treatment (>1 year after treatment)
- In the palliative / terminal phase
- Other, eg.:…………….

1. For which complaints would you want to have information about complementary care?

*(choose the options that are most important to you)*

- Nausea / vomiting
- Pain
- Sleep problems
- Anxiety
- Physical condition / fatigue
- Intestinal problems
- Lack of appetite
- Weight loss
- Weakened immune system
- Depression / “not feeling well”
- Concentration issues
- Other, eg.: ………………….

A “decision aid” is an instrument that helps to make a choice about a difficult issue. A medical decision aid can support decision making concerning treatment of a disease. A good decision aid offers patients reliable and balanced information about the pros en cons of various treatment options. Additionally it can support the process of weighing of options and taking a decision. This makes it easier to choose the treatment option that is most suitable for you.

1. Would you use a decision aid for complementary care, and how would you use it?

*(More than one answer allowed)*

- Yes, to determine the usefulness of complementary care for my child. > which type of care (question 17)
- Yes, as a source of information for a conversation with my child’s physician. I want to decide together whether complementary care is useful for my child. > which type of care (question 17)
- Yes, to read or exchange experiences with complementary care > which type of care (question 17)
- Yes, to find reliable complementary care providers > which type of care (question 17)
- Yes, to ask questions to an expert about complementary care > which type of care (question 17)
- Yes, other, eg.: > which type of care (question 17)
- No, I don’t want to use a decision aid > What is the reason for now wanting to use a decision aid? (question 16)

*In case of “I don’t want to use a decision aid” (question 15) >*

1. What is the reason for not wanting to use a decision aid? After this the alternative end text of the survey

- I don’t have interest in complementary care
- I receive sufficient information from my health care professional or nurse
- I don’t have time or room to study extra information
- Otherwise, eg.:………………

Decision aids exist in different formats. You can think of a website, an app, or a leaflet. There are decision aids mainly aiming to provide reliable information, including the pros and cons of various treatment options. This makes it easier to make a choice. Another example is a decision aid that is tailored to your personal situation and personal preferences. You are supported to weigh the pros and cons of a choice step by step. This lead to a personalized choice advise.

The next questions deal with the format of such a decision aid for complementary care.

*>All other answers at question 15 (expect answer “I don’t want to use a decision aid”)>*

1. Which format of decision aid appeals to you most?

- Computer software/website > What do you think such software or website should look like? (question 19)
- An app > What should the app look like? (question 20)
- A leaflet/information booklet > What should the leaflet/information booklet look like? (question 21)
- Otherwise, eg.: > What should this decision aid look like? (question 22)

1. What do you think such software or website should look like? > (question 22)

- Reliable information about various types of complementary care
- Support with the process of weighing pros and cons resulting in a personalized choice advise about complementary care
- Otherwise, eg.:………………

*> In case of the answer “an app”:*

1. What do you think such an app should look like? > (question 23)

- Reliable information about various types of complementary care
- Support with the process of weighing pros and cons resulting in a personalized choice advise about complementary care
- Otherwise, eg.:………………

1. What should the leaflet/information booklet look like? > (question 23)

- Reliable information about various types of complementary care
- Support with the process of weighing pros and cons resulting in a personalized choice advise about complementary care
- Otherwise, eg.:……………

*In case of “otherwise” at question 20:*

1. What do you think should such a decision aid look like? > (question 23)

- Reliable information about various types of complementary care
- Support with the process of weighing pros and cons resulting in a personalized choice advise about complementary care
- Otherwise, eg.:………………

1. In which manner should the decision aid be accessible?

*(More answers allowed)*

- From the paediatric oncologist
- General information of the hospital
- From the parent organisation VOKK
- On general websites about cancer care
- From the patient platform “complementary care”
- From the general practitioner
- From complementary professional organisations
- Otherwise, eg.:

**Alternative end text:**

You pointed out not to have interest in information about or to make use of a decision aid about complementary care for your child. Therefore this is the end of the questionnaire. When you have any remarks or suggestions, you can write these below.

This text is shown when:

- Question 13 “How important is it for you to receive information about…” after the answers “unimportant” and “very unimportant”
- After all answers on question 17 “What is the reason for not wanting to use a decision aid?”

**General end text:**

This is the end of the questionnaire. Thank you for completing it. When you have any remarks or suggestions, you can write these below.

**Invitation for focus group**

When all results of the questionnaire are collected, we would like to discuss this topic further with a number of parents. We would like to further discuss what a decision aid for complementary care for children with cancer would have to look like. This meeting will probably we conducted in June.

Are we permitted to contact you for this discussion?

If yes, could you write down your email address below?

Your email address: …………………………
