# Supplementary material for: Development of an evidence-based decision aid on complementary and alternative medicine (CAM) and pain for parents of children with cancer
Source: Support Care Cancer. 2019 Sep 6;28(5):2415–29. doi: 10.1007/s00520-019-05058-8 (PMC7083801; doi:10.1007/s00520-019-05058-8)
Supplement: Supplementary file 2 — (DOCX 33 kb) [file 520_2019_5058_MOESM2_ESM.docx]

**Online resource 2: Terms and search strings systematic literature review**Supportive Care in Cancer
Development of an Evidence-Based Decision Aid on Complementary and Alternative Medicine (CAM) for Parents of Children with Cancer.
Miek C. Jong, Inge Boers, Herman van Wietmarschen, Martine Busch, Marianne C. Naafs, Gert-Jan Kaspers, Wim J.E.Tissing.
Dr. Miek C. Jong, Mid Sweden University, Department of Health Sciences, Holmgatan 10, 851 70 Sundsvall, Sweden, email: [miek.jong@miun.se](mailto:miek.jong@miun.se)

**Pubmed search 1: #hits: 1590**

Terms:

(infan* OR newborn* OR new-born* OR perinat* OR neonat* OR baby OR baby* OR babies OR toddler* OR minors OR minors* OR boy OR boys OR boyfriend OR boyhood OR girl* OR kid OR kids OR child OR child* OR children* OR schoolchild* OR schoolchild OR school child[tiab] OR school child*[tiab] OR adolescen* OR juvenil* OR youth* OR teen* OR under*age* OR pubescen* OR pediatrics[mh] OR pediatric* OR paediatric* OR peadiatric* OR school[tiab] OR school*[tiab] OR prematur* OR preterm*)

AND

(cancer[sb])

AND

("Pain"[Mesh] OR "Pain Management"[Mesh] OR pain*[tiab] OR neuralgia*[tiab] OR arthralgia[tiab] OR analge*[tiab] OR agon*[tiab])

AND

CAM:

(Complementary Medicine [sb] OR "Mindfulness"[Mesh] OR mindful*[tiab])

**Pubmed search 2: #hits: 3452**

"Infant"[Mesh] OR infant*[tiab] OR newborn*[tiab] OR new-born*[tiab] OR perinat*[tiab] OR neonat*[tiab] OR postneonat*[tiab] OR postnat*[tiab] OR baby[tiab] OR baby*[tiab] OR babies[tiab] OR toddler*[tiab] OR minors[tiab] OR minors*[tiab] OR boy[tiab] OR boys[tiab] OR boyfriend[tiab] OR boyhood[tiab] OR girl*[tiab] OR kid[tiab] OR kids[tiab] OR "Child"[Mesh] OR child*[tiab] OR schoolchild*[tiab] OR schoolchild[tiab] OR school child*[tiab] OR adolescen*[tiab] OR "Adolescent"[Mesh] OR juvenil*[tiab] OR youth*[tiab] OR teen[tiab] OR teens[tiab] OR teenager*[tiab] OR underage*[tiab] OR under age*[tiab] OR pubescen*[tiab] OR "pediatrics"[Mesh] OR pediatric*[tiab] OR paediatric*[tiab] OR peadiatric*[tiab] OR school[tiab] OR school*[tiab] OR prematur*[tiab] OR preterm*[tiab] OR puberty[tiab] OR preschool[tiab] OR suckling*[tiab] OR picu[tiab] OR nicu[tiab] OR juvenile[tiab] OR young person*[tiab] OR young people[tiab]

AND

cancer[sb]

AND

"Pain"[Mesh] OR "Pain Management"[Mesh] OR pain*[tiab] OR neuralgia*[tiab] OR arthralgia[tiab] OR analge*[tiab] OR agon*[tiab]

AND

"Complementary Therapies"[Mesh] OR complementary therap*[tiab] OR alternative medic*[tiab] OR complementary medic*[tiab] OR alternative therap*[tiab] OR "Acupuncture Therapy"[Mesh] OR "Acupuncture"[Mesh] OR acupuncture[tiab] OR "Anthroposophy"[Mesh] OR anthroposoph*[tiab] OR "Holistic Nursing"[Mesh] OR "Holistic Health"[Mesh] OR holistic[tiab] OR "Homeopathy"[Mesh] OR homeopath*[tiab] OR "Medicine, Traditional"[Mesh] OR traditional medicine[tiab] OR traditional chinese medicine[tiab] OR tcm[tiab] OR chinese traditional medicine[tiab] OR ayurved*[tiab] OR "Mind-Body Therapies"[Mesh] OR mind-body[tiab] OR body-mind[tiab] OR "Aromatherapy"[Mesh] OR aromatherap*[tiab] OR aroma therap*[tiab] OR "Hypnosis"[Mesh] OR hypnos*[tiab] OR hypnot*[tiab] OR "Meditation"[Mesh] OR meditat*[tiab] OR "Mental Healing"[Mesh] OR mental heal*[tiab] OR "Relaxation Therapy"[Mesh] OR relaxation therap*[tiab] OR relaxing therap*[tiab] OR "Therapeutic Touch"[Mesh] OR therapeutic touch*[tiab] OR reiki[tiab] OR "Yoga"[Mesh] OR yoga[tiab] OR osteopath*[tiab] OR "Chiropractic"[Mesh] OR "Manipulation, Chiropractic"[Mesh] OR chiropractic*[tiab] OR "Kinesiology, Applied"[Mesh] OR kinesiolog*[tiab] OR "Mindfulness"[Mesh] OR mindful*[tiab] OR "Phytotherapy"[Mesh] OR reflexotherap*[tiab] OR reflexo therap*[tiab] OR reflextherap*[tiab] OR reflex therap*[tiab] OR "Art Therapy"[Mesh] OR art therap*[tiab] OR color therap*[tiab] OR colour therap*[tiab] OR dance therap*[tiab] OR music therap*[tiab] OR play therap*[tiab] OR spiritual therap*[tiab] OR "Acoustic Stimulation"[Mesh] OR acoustic stimulat*[tiab] OR "Orthomolecular Therapy"[Mesh] OR orthomolecular therap*[tiab] OR vitamin therap*[tiab] OR megavitamin therap*[tiab] OR "Diet Therapy"[Mesh] OR "diet therapy"[Subheading] OR diet therap*[tiab] OR "Probiotics"[Mesh] OR probiotic*[tiab] OR "Dietary Supplements"[Mesh] OR supplement[tiab] OR supplements[tiab] OR "Functional Food"[Mesh] OR superfood*[tiab] OR super food*[tiab] OR food supplement*[tiab] OR dietary supplement*[tiab] OR detox*[tiab] OR "Naturopathy"[Mesh] OR naturopath*[tiab] OR naturo path[tiab] OR naturo paths[tiab] OR massage*[tiab] OR "Herbal Medicine"[Mesh] OR herbal medic*[tiab] OR herbal therap*[tiab] OR herb[tiab] OR herbs[tiab] OR "Mistletoe"[Mesh] OR mistletoe*[tiab] OR "Viscum"[Mesh] OR viscum[tiab] OR additional therap*[tiab] OR supporting therap*[tiab] OR support therap*[tiab] OR supportive therap*[tiab] OR "Combined Modality Therapy"[Mesh] OR combination therap*[tiab] OR "Faith Healing"[Mesh] OR faith heal*[tiab] OR "Behavior Therapy"[Mesh] OR behavior therap*[tiab] OR behaviour therap*[tiab] OR behavioral intervention*[tiab] OR behavioural intervention*[tiab] OR supportive care[tiab] OR supportive caring[tiab] OR body-based[tiab] OR "Food"[Mesh] OR food[tiab] OR foods[tiab] OR feeding[tiab] OR raw food*[tiab] OR "Spirituality"[Mesh] OR spiritual*[tiab] OR "Religion"[Mesh] OR "Religion and Medicine"[Mesh] OR religio*[tiab] OR Electroacupuncture[tiab] OR Moxibustion[tiab] OR Auriculotherap*[tiab] OR Biofeedback[tiab] OR Breathing Exercise*[tiab] OR guided Imagery[tiab] OR Tai Ji[tiab] OR Acupressure*[tiab]

**CINAHL search #hits: 144**

(MH "Neoplasms+") OR (TI,AB cancer* OR neoplas* OR oncolog* OR malignan* OR tumo?r* OR carcinoma* OR adenocarcinoma* OR sarcoma* OR leuk?emia OR chemotherap*)

AND

(MH "Child+") OR (MH "Pediatrics+") OR (TI,AB infan* OR newborn* OR new-born* OR perinat* OR neonat* OR baby OR baby* OR babies OR toddler* OR minors OR minors* OR boy OR boys OR boyfriend OR boyhood OR girl* OR kid OR kids OR child OR child* OR children* OR schoolchild* OR schoolchild OR school child OR school child* OR adolescen* OR juvenil* OR youth* OR teen* OR under*age* OR pubescen* OR pediatrics OR pediatric* OR paediatric* OR peadiatric* OR school OR school* OR prematur* OR preterm*)

AND

(MH "Alternative therapies+") OR (MH "Mindfulness") OR (TI,AB alternative therap* OR mindful*)

AND

(MH "Pain+") OR (TI,AB neuralgia* OR arthralgia OR analge* OR agon* )

Cochrane search #hits: 1347

KIND (241287)

(infant OR infan* OR newborn OR newborn* OR new-born* OR baby OR baby* OR babies OR neonat* OR childOR child* OR schoolchild* OR schoolchild OR school child OR school child* OR kid OR kids OR toddler* OR adolescent OR adoles* OR teen*OR boy* OR girl* OR minors OR minors* OR underag* OR under ag* OR juvenil* OR youth*OR kindergar* OR puberty OR puber* OR pubescen* OR prepubescen* OR prepuberty* OR pediatrics OR pediatric* OR paediatric* OR peadiatric* OR schools OR nursery school* OR preschool* OR pre school* OR primary school* OR secondary school* OR elementary school* OR elementary school OR high school* OR highschool* OR school age OR schoolage OR school age* OR schoolage* OR infancy)

AND KANKER (129730)

1. MeSH descriptor: [Neoplasms] explode all trees

2. cancer* or neoplas* or oncolog* or malignan* or tumor* or tumour* OR carcinoma* OR adenocarcinoma* or sarcoma* or leukemia or leukaemia:ti,ab,kw

3. #1 or #2

AND PAIN (125536)

1. MeSH descriptor: [Pain] explode all trees

2. MeSH descriptor: [Pain Management] explode all trees

3. pain* or neuralgia* or arthralgia or analge* or agon*:ti,ab,kw

4. #1 or #2 or #3

AND CAM (38865)

1. MeSH descriptor: [Complementary Therapies] explode all trees

2. MeSH descriptor: [Mindfulness] explode all trees

3. Complementary therap* or complementary medicine or alternative therap* or
 alternative medicine

4. #1 OR #2 OR #3

**Embase search: #hits: 1993**

1. infant/ or infancy/ or newborn/ or baby/ or child/ or preschool child/ or school child/ or adolescent/ or juvenile/ or boy/ or girl/ or puberty/ or prepuberty/ or pediatrics/ or primary school/ or high school/ or kindergarten/ or nursery school/ or school/

2. (infant* or newborn* or new born* or baby or baby* or babies or neonate* or child* or school child* or schoolchild* or school age* or schoolage* or pre school* or preschool* or kid or kids or toddler* or adoles* or teen* or boy* or girl* or minors* or under ag* or underage* or juvenil* or youth* or puber* or pubescen* or prepubescen* or prepubert or pediatric* or paediatric* or peadiatric* or school or schools or high school* or highschool* or primary school* or nursery school* or elementary school or secondary school* or kindergar*).ti,ab,kw.

3. 1 or 2

4. exp neoplasms/ or (cancer* or neoplas* or oncolog* or malignan* or tumo?r* or carcinoma* or adenocarcinoma* or sarcoma* or leuk?emia or chemotherap*).ti,ab,kw.

5. exp pain/ or (pain* or neuralgia* or arthralgia or analge* or agon*).ti,ab,kw.

6. exp alternative medicine/ or exp integrative medicine/ or exp psychosomatics/ or exp acupuncture/ or exp traditional medicine/ or exp hypnosis/ or exp meditation/ or exp herb/ or exp animal assisted therapy/ or exp holistic nursing/ or exp homeopathy/ or exp aromatherapy/ or exp meditation/ or exp relaxation training/ or exp yoga/ or exp chiropractic/ or exp kinesiology/ or exp mindfulness/ or exp phytotherapy/ or exp art therapy/ or exp auditory stimulation/ or exp diet therapy/ or probiotic agent/ or exp diet supplementation/ or exp functional food/ or exp herbal medicine/ or exp viscum/ or exp spiritual healing/ or exp behavior therapy/ or exp food/ or exp religion/

7. ("integrative medicine" or orthomolecular or "selenium vitamin e select" or mind-body or mindful* or yoga or ayurved* or "ayur veda" or "ayur vedic" or siddha or guggal or guggul or "balint group*" or cyberbiology or "flower remedies" or (bach and flower) or "flower essences" or heliotherapy or cayce or sophrology or "laughter therapy" or meditation or kundalini or hypnosis or hypnotherapy or simonton or biofeedback or neurofeedback or "self-help groups" or "support groups" or "autogenic training" or "transpersonal psychology" or "relaxation therapy" or "relaxation techniques" or psychoneuroimmunology or "psychosomatic medicine" or "aversion therapy" or "art therapy" or "music therapy" or "dance therapy" or "drama therapy" or dramatherapy or psychodrama or journaling or "animal assisted therapy" or "feng shui" or syntonic or "mental healing" or "spiritual healing" or "alternative healer*" or "faith heal*" or "special healer*" or shamanism or shaman or santeria or witchcraft or voodoo or magic or prayer* or praying or spirituality or psychospiritual or "holistic nursing" or "pastoral care" or "alcoholics anonymous" or kirlian or "ear candle*" or "native american traditional medicine" or "korean traditional medicine" or "chinese traditional medicine" or "chinese medicine" or "oriental traditional medicine" or "east asian traditional medicine" or "kampo medicine" or "tibetan traditional medicine" or qi or ch'i or yin-yang or acupuncture or electroacupuncture or auriculoacupuncture or auriculotherapy or "dry needling" or "trigger point injection*" or "transcutaneous electric nerve stimulation" or acumoxa or "fu zheng" or "huangdi neijing" or moxibustion or "qi gong" or qigong or "tai chi" or aikido or "traditional medicine" or "primitive medicine" or "folk medicine" or folklore or "traditional indigenous" or "home remedies" or "home remedy" or "traditional healer*" or unani or tibbi or tibb or "traditional african medicine" or curanderismo or "tibetan medicine" or kampo or "arabic medicine" or la'au or yoruba or homeopath* or homoeopath* or "materia medica" or "environmental medicine" or "restricted environmental stimulation therapy" or radiesthesia or naturopath* or anthroposophy or eclecticism or kneipp or phytotherapy or "chinese herb*" or "herbal drugs" or "herbal medicine" or "medicinal plants" or "plant extracts" or botanicals or herbal or herbals or herbalism or "phytogenic antineoplastic agents" or phytoestrogen* or pharmacognosy or "herb-drug interactions" or "artichoke extract" or "black cohosh" or "ginkgo biloba" or bilobalides or ginkgolides or hypericum or (st and john and wort) or garlic or (allicin and "sulfinic acids") or ginseng or panax or "astragalus plant" or "golden seal" or goldenseal or glyconutrient* or pomegranate or (honey and therapy) or guarana or acai or "ba wei di huang wan" or bear-bile or berberine or "borage oil" or arnica or "cetyl myristoleate" or "citrus pectin" or cordyceps or echinacea or "flax seed" or "saw palmetto" or "urtica dioica" or kava or hawthorn or "horse chestnut seed" or "witch hazel" or bilberry or ginger or garcinia or "aloe vera" or capsicum or feverfew or "green tea" or "tea tree oil" or yohimbe or yohimbine or rhodiola or valerian or heliotrope or "bee pollen" or myrrh or "cat's claw" or "uncaria tomentosa" or "evening primrose" or "dong quai" or fenugreek or maitake or "grifola frondosa" or marshmallow or "mulberry leaf extract" or "olive leaf extract" or "pine bark extract" or (hoodia and appetite) or psyllium or "stanol therapy" or tumeric or turmeric or curcumin or mistletoe or iscador or "mahonia aquifolium" or (oleum and menthae and piperitae) or "thunder god vine" or "peppermint oil" or "milk thistle" or "silybum marianum" or silymarin or silybin or "ma huang" or "purple sweet potato anthocyanin" or ephedra or padma or hoxsey or essiac or kombucha or pau or pc-spes or pygeum or "red yeast rice" or sam-e or tinospora or phyllanthus or nutriceutical* or nutraceutical* or "diet fads" or food or food or "vegetarian diet" or vegetarianism or vegan or vegans or veganism or wssf or "water-soluble soybean fiber" or phytosterols or phytosterol or isoflavones or isoflavone or ipriflavone or genistein or "coley toxins" or "compound 714-X" or antineoplastons or "edetic acid" or edta or "ethylenediaminetetraacetic acid" or immunoaugmentative or revici or "cell therapy" or organotherapy or "tissue therapy" or dibella or "greek cancer cure" or krebiozen or laetrile or "urine therapy" or reishi or "staphage lysate" or cellasene or "chelation therapy" or "chitosan weight loss" or apitherap* or "neural therapy" or prolotherapy or neuraltherapy or electrodiagnostics or iridolog* or iridodiagnosis or bioresonance or "expressive writing" or neuroacoustic or revici or naprapathy or "spinal manipulation" or chiropractic or massage or "osteopathic medicine" or tuina or shiatsu or reflexology or reflexotherapy or rolfing or "applied kinesiology" or "energy polarity healing" or "pranic healing" or feldenkrais or "applied kinesiology" or pilates or "post isometric contract" or "hold relax" or effleurage or "myofascial release" or "gravity inversion" or hellerwork or hippotherapy or hydrotherapy or balneotherapy or balneology or ammotherapy or "sand bath*" or "finnish baths" or "mud pack*" or "mud therapy" or "mud bath*" or thalassotherapy or climatotherapy or speleotherapy or "manual lymphatic drainage" or "manual lymph drainage" or mesotherapy or myotherapy or "neurocranial restructuring" or "neuromuscular integrated action" or reichian or petrissage or watsu or "colour therapy" or "color therapy" or aromatherapy or "heat therapy" or electrotherap* or holotropic or "alternate nostril breathing" or "unilateral forced nostril breathing" or biofield or "healing art" or "therapeutic touch" or "healing touch" or "caring touch" or reiki or huna or "external qi healing" or "psychic healing" or "sensory deprivation" or chronotherapy or vitalism or bioelectromagnetic* or biomagnets or bioelectromagnetism or "transcutaneous electrical nerve stimulation" or "repetitive transcranial magnetic stimulation" or trigger-point-therapy or "myofascial trigger point pain electrotherapy" or bobath or electrostimulation or "blue light treatment" or "holographic repatterning" or thermochemoradiotherapy or "infrasound therapy" or "ion generated" or "ion generating" or "ion generator" or nccam or nccih).ti,ab,kw.

8. 6 or 7

9. (megados* or "high dosage*" or "high dose" or "large dosage*" or "large dose*" or "large dosed" or adjuvant or unorthodox or unconventional or supplement* or "high daily intake").ti. and (vitamin* or vegetables or mineral or androstenedione or andro or "arachidonic acid" or "ascorbic acid" or biotin or boron or bromelain* or calcium or caroten* or choline or chromium or "colloidal silver" or "conjugated linoleic acid" or copper or "docosahexaenoic acid" or dha or "epigallocatechin gallate" or EGCG or "folic acid" or inositol or iodine or iron or l-arginine or magnesium or manganese or molybdenum or niacin or niacinamide or "phosphatidyl choline" or lecithin or potassium or pyridoxine or riboflavin or retinoid* or retinoic or retinal or selenium or silicon or thiamine or tocopherol* or vanadium or zinc or phytomedicine or micronutrient*).ti,ab,kw.

10. ((adjuvant or complementary or unorthodox or unconventional or supplement*) and ("co-enzyme Q10" or ubiquinone or carnitine or glutamine or phenylalanine or "glucosamine sulfate" or "chondroitin sulfate*" or "lipoic acid" or "medium chain triglycerides" or phosphatidylserine or melatonin or dhea or taurine or lysine or tyrosine or gamma-oryzanol or "fatty acids" or "amino acids" or s-adenosylmethionine)).ti.

11. ("eye movement" and (desensitization or emdr)).ti,ab,kw.

12. ((mental or guided or visualization or rehearsal) and imagery).ti,ab,kw.

13. ("group psychotherapy" and cancer).ti,ab,kw.

14. ("progressive relaxation" and (pain or anxiety)).ti,ab,kw.

15. ("pet therapy" and animal*).ti,ab,kw.

16. (religion and medicine).ti,ab,kw.

17. ((aboriginal or aborigines) and (medicinal or medicine)).ti,ab,kw.

18. ((wholistic or holistic) and (health or medicine or nursing)).ti,ab,kw.

19. (bowen and family and (therapy or systems or technique)).ti,ab,kw.

20. (botanical and (supplementary or supplemental or medicine or medicinal or remedy or remedies or therapy or therapeutic or management or treatment or antineoplastic* or preparation*)).ti,ab,kw.

21. (oil and "bitter orange").ti,ab,kw.

22. ((bromelain or pineapple) and (therapy or therapeutic or treatment or anti-inflammatory or antiinflammatory)).ti,ab,kw.

23. (flax and (tumors or cancerc or "plant extracts")).ti,ab,kw.

24. ((cannabis or marijuna or marihuana) and (medical or "therapeutic use" or antiinflammatory or "oral administration")).ti,ab,kw.

25. (resveratrol and (therapy or therapeutic)).ti,ab,kw.

26. (extract* and (butterbur or butter bur or "grape seed")).ti,ab,kw.

27. ("human growth hormone" and aging).ti,ab,kw.

28. ((5-Hydroxytryptophan or "alpha-lipoic acid") and "therapeutic use").ti,ab,kw.

29. (cinnamon and (antioxidants or oils)).ti,ab,kw.

30. (clove and (oils or extract* or infusion)).ti,ab,kw.

31. ((chamomile or cranberry) and "plant extracts").ti,ab,kw.

32. (licorice and (root or roots)).ti,ab,kw.

33. (lavender and (oil or oils or odors)).ti,ab,kw.

34. (mushroom* and (medicinal or therapy or antifungal or antineoplastic)).ti,ab,kw.

35. (palo and arco).ti,ab,kw.

36. ((avellanedae or impetiginosa) and tabebuia).ti,ab,kw.

37. ("pumpkin seed*" and (therapy or treatment)).ti,ab,kw.

38. ("rice bran" and (diet or dietary)).ti,ab,kw.

39. ((probiotics or prebiotics or synbiotics or health food or "functional food*") and therapy).ti,ab,kw.

40. (macrobiotic and diet).ti,ab,kw.

41. ((pritikin or mcdougall or gerson or kelley or fasting) and "diet therapy").ti,ab,kw.

42. ("proteolytic enzymes" and ("diet therapy" or "oral administration") and cancer).ti,ab,kw.

43. ((wigmore or livingston-wheeler or atkins or low-carb or low-carbohydrate or diamond or "high fiber" or "high fibre" or macrobiotic or ketogenic or mediterranean or paleolithic or asian or ornish) and diet).ti,ab,kw.

44. ((omega-3 or "fish oil" or n3 or n-3) and (oil or oils or PUFA or "fatty acid*") and (diet or dietary or supplement*)).ti,ab,kw.

45. ((diet or dietary) and cultural).ti,ab,kw.

46. ((fruit or fruits or plant or plants or soy or soybean*) and juice and (prevention or therapy)).ti,ab,kw.

47. ((anti-oxidant* or antioxidant* or consumption or treatment or therapy or prevention or therapeutic) and (flavonoid* or bioflavinoid* or bioflavonoid*)).ti,ab,kw.

48. (antioxidants and blueberr*).ti,ab,kw.

49. (creatine and "huntington disease" and (therapy or therapeutic)).ti,ab,kw.

50. (shark and cartilage).ti,ab,kw.

51. (powdered and cartilage and wound).ti,ab,kw.

52. ((therapeutic or prevention or therapy) and shiitake).ti,ab,kw.

53. (stevia and sweetener).ti,ab,kw.

54. stevia.ti.

55. ((intravenous and ozone and therapy) or (ozonotherapy or "ozone therapy" or oxymedicine or "hyperoxigenation therapy")).ti,ab,kw.

56. ("hydrogen peroxide" and (intravenous or infusions) and (dosage or therapy or "therapeutic use")).ti,ab,kw.

57. (("hyperbaric oxygen therapy" or "hyperbaric oxygenation") not (wounds or "wound healing" or "decompression sickness" or diving)).ti,ab,kw.

58. ("t tn" and vaccinat*).ti,ab,kw.

59. (coffee and enema*).ti,ab,kw.

60. ("hydrazine sulfate" and (therapy or "antineoplastic agents") and cancer).ti,ab,kw.

61. ((livingston or maggot) and therapy).ti,ab,kw.

62. ((cancer or neoplasms) and pannon).ti,ab,kw.

63. (chacon and cancer).ti,ab,kw.

64. (("noni juice" or "morinda citrifolia" or mth-68) and ("antineoplastic agents" or cancer or analgesic or antihelminthic)).ti,ab,kw.

65. ((treatment or therapy) and insulin and potentiation).ti.

66. (("thymus extract" or "pancreatic extract") and "therapeutic use").ti,ab,kw.

67. (mora and therapy).ti,ab,kw.

68. (maruyama and vaccine).ti,ab,kw.

69. ((manipulative or manipulation) and osteopathic).ti,ab,kw.

70. (tui and na).ti,ab,kw.

71. (("body centered" or "body oriented") and psychotherapy).ti,ab,kw.

72. ((approach or psychophysical or bodywork or body) and trager).ti,ab,kw.

73. (alexander and technique).ti,ab,kw.

74. (amma and therapy).ti,ab,kw.

75. (conductive and education).ti,ab,kw.

76. ((craniosacral or cranio-sacral) and (therapy or treatment)).ti,ab,kw.

77. (jin and shin).ti,ab,kw.

78. (hawaiian and lomi).ti,ab,kw.

79. (meridian and therapy).ti,ab,kw.

80. (saline and "nasal irrigation").ti,ab,kw.

81. (natural and healing).ti.

82. (psychic and surgery).ti,ab,kw.

83. (polarity and therapy).ti,ab,kw.

84. ((ultrasound or radiofrequenc*) and "induced hyperthermia").ti,ab,kw.

85. ((magnet or electromagnetic) and therapy).ti.

86. ((therapy or treatment) and "millimeter wave*").ti,ab,kw.

87. 8 or 9 or 10 or 11 or 12 or 13 or 14 or 15 or 16 or 17 or 18 or 19 or 20 or 21 or 22 or 23 or 24 or 25 or 26 or 27 or 28 or 29 or 30 or 31 or 32 or 33 or 34 or 35 or 36 or 37 or 38 or 39 or 40 or 41 or 42 or 43 or 44 or 45 or 46 or 47 or 48 or 49 or 50 or 51 or 52 or 53 or 54 or 55 or 56 or 57 or 58 or 59 or 60 or 61 or 62 or 63 or 64 or 65 or 66 or 67 or 68 or 69 or 70 or 71 or 72 or 73 or 74 or 75 or 76 or 77 or 78 or 79 or 80 or 81 or 82 or 83 or 84 or 85 or 86

88. 3 and 4 and 5 and 87
