# Supplementary material for: Development of an evidence-based decision aid on complementary and alternative medicine (CAM) and pain for parents of children with cancer
Source: Support Care Cancer. 2019 Sep 6;28(5):2415–29. doi: 10.1007/s00520-019-05058-8 (PMC7083801; doi:10.1007/s00520-019-05058-8)
Supplement: Supplementary file 3 — (DOCX 24.7 kb) [file 520_2019_5058_MOESM3_ESM.docx]

**Online resource 3: Interview guide clinical experts**Supportive Care in Cancer
Development of an Evidence-Based Decision Aid on Complementary and Alternative Medicine (CAM) for Parents of Children with Cancer.
Miek C. Jong, Inge Boers, Herman van Wietmarschen, Martine Busch, Marianne C. Naafs, Gert-Jan Kaspers, Wim J.E.Tissing.
Dr. Miek C. Jong, Mid Sweden University, Department of Health Sciences, Holmgatan 10, 851 70 Sundsvall, Sweden, email: [miek.jong@miun.se](mailto:miek.jong@miun.se)

1. How do you deal with the pain of children with cancer? What are the most important elements in the treatment of or approaches towards children with cancer and pain (and their parents)?
2. Tell about your experiences with complementary care (CAM) for pain of children with cancer?

- In general: with which CAM options do you have good experiences (and why), with which not?
- During specific stages of the disease and/or treatment?
- For particular groups of children (eg. age groups, various types of cancer, at home or in the hospital)?
- Are there different experiences with procedural pain versus general pain with cancer (treatment), if yes, which?

1. What are the side effects and/or interactions of CAM modalities for treatment of pain in children with cancer?
2. Tell about your experiences on CAM with regard to the most common complaints from children with cancer?
   1. Physical conditions / fatigue
   2. Anxiety
   3. Pain
   4. Suppressed immune system
   5. Nausea / vomiting
   6. Depression / uncomfortable feeling
   7. Reduced appetite
   8. Bowel issues
   9. Concentration issues
   10. Weight loss
3. Which treatment options do you consider part of CAM?
   - E.g. cognitive behaviour therapy, hypnotherapy, distraction, music therapy, creative therapy, imagination, relaxation therapy, breathing therapy
4. Which policy / protocol / guidelines do you use regarding CAM?
5. What is the most promising “clinical practise case” regarding CAM in your experience?
   - Type of CAM?
   - Specific complaints?
   - Target group?
