# Supplementary material for: Development of an evidence-based decision aid on complementary and alternative medicine (CAM) and pain for parents of children with cancer
Source: Support Care Cancer. 2019 Sep 6;28(5):2415–29. doi: 10.1007/s00520-019-05058-8 (PMC7083801; doi:10.1007/s00520-019-05058-8)
Supplement: Supplementary file 4 — (DOCX 23.6 kb) [file 520_2019_5058_MOESM4_ESM.docx]

**Online resource 4: Questionnaire for evaluation website**Supportive Care in Cancer
Development of an Evidence-Based Decision Aid on Complementary and Alternative Medicine (CAM) for Parents of Children with Cancer.
Miek C. Jong, Inge Boers, Herman van Wietmarschen, Martine Busch, Marianne C. Naafs, Gert-Jan Kaspers, Wim J.E.Tissing.
Dr. Miek C. Jong, Mid Sweden University, Department of Health Sciences, Holmgatan 10, 851 70 Sundsvall, Sweden, email: miek.jong@miun.se

1. What is your opinion about the feel and look?

2. What is your opinion about the content structure?

3. Can you easily navigate and find the information?

4. What is your opinion on the readibility of the text?

5. How is your understanding of the content?

6. Which parts or content is informative?

7. Which parts or content is abundant or not needed?

8. What do you miss?

9. What other remarks or suggestions do you have?
