# Supplementary material for: Development of an evidence-based decision aid on complementary and alternative medicine (CAM) and pain for parents of children with cancer
Source: Support Care Cancer. 2019 Sep 6;28(5):2415–29. doi: 10.1007/s00520-019-05058-8 (PMC7083801; doi:10.1007/s00520-019-05058-8)
Supplement: Supplementary file 5 — (DOCX 45 kb) [file 520_2019_5058_MOESM5_ESM.docx]

**Online resource 5: Overview other studies analysed (N=18)**Supportive Care in Cancer
Development of an Evidence-Based Decision Aid on Complementary and Alternative Medicine (CAM) for Parents of Children with Cancer.
Miek C. Jong, Inge Boers, Herman van Wietmarschen, Martine Busch, Marianne C. Naafs, Gert-Jan Kaspers, Wim J.E.Tissing.
Dr. Miek C. Jong, Mid Sweden University, Department of Health Sciences, Holmgatan 10, 851 70 Sundsvall, Sweden, email: miek.jong@miun.se

| **Study** | **Design** | **Sample** | **Intervention(s)** | **Findings** | **Findings short**** | **Quality of evidence** | **Quality level** |
| --- | --- | --- | --- | --- | --- | --- | --- |
| **Hypnotherapy** | | | | | | | |
| Zeltzer 1982 | Randomized, not controlled intervention study, evaluating **hypnotherapy** versus nonhypnotic techniques for pain associated with BMA or LP | Children and adolescents (6-17 years) with cancer undergoing BMA or LP (n=33) | **Hypnotherapy:** deep breaths, practice sessions, individual imagery and fantasy. Ending with a post-hypnotic suggestion. Following that, children were taught self-hypnosis.  Nonhypnotic techniques: nonhypnotic behavioral techniques (combination of deep breathing, distraction, and practice sessions) | -Hypnoses and non-hypnotic techniques were associated with an overall reduction in pain for BMA/ LP  -Hypnosis was more effective than non-hypnotic techniques in reducing self-reported pain for BMA/ LP. | *Hypnosis vs nonhypnotic techniques*  + for self-reported pain  *Post treatment vs baseline*  + for self-reported pain (both groups) | Randomized study, no control group, sufficient sample size. Not reported if allocation was known by the researcher at forehand or how both groups were matched for age and disease. No blinding, no selective reporting, appropriate analysis, study completed as planned, no missing data. | Moderate |
| Kuttner 1988 | RCT, evaluating **hypnotherapy** versus distraction and standard care for distress, pain and anxiety associated with BMA | Children (3-10 years) with acute lymphoblastic leukemia or acute myeloblastic leukemia who according to medical staff needed help in managing BMA (n=48) | **Hypnotherapy:** indirect suggestions related to stories and adventures, individually tailored. Also direct hypnotic techniques such as the “pain switch”. Parents were included in the sessions.  Distraction: including elements as: showing physical objects (toys, puppets, pop-up books), distracting questions, physical activities (blowing a bubble or squeezing parents hands, deep breathing). Parents were included in the sessions.  Standard care: current standard medical practice | *At first intervention*  **In the older age group (7-10 years):**  -Lower observed pain and anxiety, as well for hypnotherapy as for distraction versus standard care.  -Distraction is better in reducing observed and self-reported pain then hypnotherapy.  **In the younger age group (3-6,5 years):**  -Lower observed and self-reported pain, as well for hypnotherapy as for distraction versus standard care.  -Hypnotherapy is better in reducing observed and self-reported pain then distraction  *At second intervention:*  -All groups showed reductions and the control group appeared to be contaminated.  -The hypnotic method with its internal focus had an all-or-none effect, whereas distraction appeared to require that coping skills be learned over one session or more.  -Distraction seems to be more age appropriate in older children | *Hypnosis or distraction vs standard care:*  + for pain in older and younger children  *Hypnosis vs distraction:*  + for pain in older children with distraction  + for pain in younger children with hypnosis | RCT, sufficient sample size. Lack in blinding, independent observers, inter-rater reliability was tested and found to be good, possible bias in the control group, who at the second intervention also showed clinical effects, due to learning effects of staff. No selective reporting, appropriate analysis, 48 started the study, and 30 finished, so drop-out of 18 patients, due to several reasons (death, not returned for BMA, etc.) | Low |
| Wall 1989 | RCT, evaluating **hypnotherapy** versus active cognitive coping strategies for pain and anxiety associated with BMA or LP | Children (6-18 years), hematology and oncology patients undergoing BMA or LP (n=20) | **Hypnotherapy:** Hypnotic induction, progressing from relaxation to visual imagery.  Cognitive coping: Choice from 4 activities designed to cause a shift in attention during BMA or LP. | -No differences in pain reduction by treatment strategy.  -Both treatment strategies had better scores post treatment in: self-reported pain, observer-reported pain, child self-reported pain relief, McGill Pain Questionnaire scores and MPQ pain rating index.  -Neither technique was effective in anxiety reduction.  -Hypnotizability scale scores failed to correlate with degree of pain reduction. | *Hypnosis vs cognitive coping:*  = no differences between groups.  *Post treatment vs baseline (both groups):*  ++ for self-reported pain  ++for observer-reported pain  + for child self-report pain relief  + for McGill Pain Questionnaire  + for MPQ pain rating index  = for anxiety | RCT, small sample size. Observers, trainers and experimenters were blind to treatment allocation, patients were not informed that hypnotherapy was one of the strategies, similar attention was given to both groups. No description of monitoring compliance of exercises. No selective reporting, appropriate analysis, study completed as planned, no missing data. | High |
| Liossi 1999 | RCT evaluating **hypnosis** and CBT versus standard care for pain and distress associated with BMA | Children and adolescents (5–15 years old) with leukemia undergoing BMAs (n=30) | **Hypnosis:** visual imagery (favorite place, activity, or television program), relaxation techniques, progressive muscle relaxations and autogenic relaxation. “Analgesic suggestions” as request for numbness, topical, local and glove anesthesia  CBT: including relaxation training, breathing exercises and cognitive restructuring  Standard care: a standard lidocaine injection, the same as the children in the intervention groups | -Hypnosis and CBT are both more effective for reducing pain and anxiety as compared to standard care.  -Hypnosis and CBT are comparable for pain reduction, but less behavioral distress observed in hypnosis group | *Hypnosis vs standard care:*  ++ for self-reported pain  ++ for observed distress  *CBT vs standard care:*  ++ for self-reported pain  ++ for observed distress  *Hypnosis vs CBT:*  = no difference for self-reported pain  ++ for observed distress | RCT, small sample size, all measures that could be blinded, were blinded. Independent observer and doctor were blinded to allocation of treatment. Involvement of parents and presence of therapists during BMA were the same in all three groups, inter-rater reliability was tested and found to be good, self-reported findings mirrored the observed findings. Selective reporting, appropriate analysis, study completed as planned, no missing data | Moderate |
| Hawkins 1998 | Randomized, not controlled intervention study evaluating **direct hypnotherapy** versus **indirect hypnotherapy** for pain and anxiety associated with LP | Children and adolescents (6-16 years old) with leukemia or non-Hodgkin lymphoma undergoing LPs (n=30) | **Direct hypnotherapy:** direct hypnotic suggestions were all directed towards imagining numbness, topical and local, glove anesthesia and the switchbox. Directed by therapist.  **Indirect hypnotherapy**: The setting sun metaphor and the Mexican food metaphor were used for indirect suggestions. Directed by therapist. | -Both groups had significantly reduced self-reported pain and anxiety and observer-reported distress, during LP with hypnosis as compared to baseline  -There were no significant differences between types of hypnotic intervention (direct vs indirect)  -Higher level of hypnotizability was associated with increased treatment benefit for self-reported pain, anxiety, and observer-rated distress | *Direct vs indirect hypnosis*  = no differences in pain, anxiety and distress  *Post treatment vs baseline:*  + for self-reported pain  + for anxiety  + for observer-reported distress | Randomized study, no control group, sufficient sample size, independent observer, inter-rater reliability was tested and found to be good, both self-assessment outcomes of children as well as independent observer evaluations mirrored each other with respect to changes in outcome. Lack of blinding, no selective reporting, correct analysis, study completed as planned, no missing data. | Low |
| Hilgard 1982 | Observational study on hypnotherapy induced relief of anxiety and pain associated with BMA | Children and adolescents (6-19 years) with cancer, chiefly forms of leukemia, undergoing repeated BMAs (n=24) | **Hypnotherapy:** imaginative exercises such as blowing out candles. Indirect suggestions. | -Post-treatment: self-reported pain and observer-rated pain were diminished.  -No difference between self-reported and observed pain for patients under age 10.  -For children age 10 and older there was a difference between self-reported and observed pain  -There were minor but significant sex differences both in observed pain and in self-reported pain, with the females reporting more pain. | *Post treatment vs baseline:*  + for self-reported pain  ++ for observer-rated pain | Observational, no control group, sufficient sample size, no blinding, selective reporting, appropriate analysis, study completed as planned, no missing data. | Low |
| Kellerman 1983 | Prospective observational study on the effects of individualized hypnotherapy on discomfort and anxiety associated with BMAs, LPs and chemotherapeutic injections | Adolescents (mean 14 years) with various types of cancer undergoing BMAs, LPs and chemotherapeutic injections, referred by their oncologists because of procedural distress (n=18) | **Hypnotherapy:** individualized, suggestions for progressive muscular relaxation, slow rhythmic breathing, favorite place hypnotic induction. Posthypnotic suggestions for increased well-being, reduced discomfort, and greater mastery during the procedure were given. Following that, children were taught self-hypnosis. | -Significant reductions in pain, anxiety and multiple measures of distress after hypnosis training.  -Pre-intervention data showed no pattern of spontaneous remission or habituation, and, in fact, an increasing anticipatory anxiety was observed before hypnotic treatment.  -A non-significant trend toward greater self-esteem was present. The predicted changes in the Locus of Control and General Illness Impact were not found.  -Comparisons between hypnosis rejectors and successful users unusually showed higher levels of pretreatment anxiety in the former. | *Post treatment vs baseline:*  + for pain before painful procedure,  ++ for pain during painful procedure  ++ for pain after painful procedure  + for anxiety and distress | Observational, no control group, small sample size, heterogonous group, no blinding, authors applied hypnotherapy themselves, selective reporting, appropriate analysis, 2 patients rejected hypnotherapy (religious, feeling uncomfortable), outcome measures are nor well defined. | Low |
| **MIND-BODY (including imagery, meditation, breathing techniques)** | | | | | | | |
| Pederson 1996 | Pretest and posttest with randomized control group design, with an extension in which the control group experienced the intervention following posttest. To evaluate the effect of the effect of teaching children and their parents about selected **non-pharmacologic techniques** during LPs for pain and distress. | Children (6-14 years) with acute leukocytic leukemia undergoing LPs (n=8) | Mind-body teaching program: Parent-child program based on distraction, **breathing, relaxing, imagery**, changing perceptions of painful stimuli using videotape, a booklet and distraction materials and a support person during LP  Control group: standard care, afterwards the same program as experimental group, though starting a LP later | -No differences between groups for distress and self-reported pain.  -The treatment group had: fewer expressions of verbal resistance, fewer instances of muscular rigidity and more instances of parental interventions.  -Both groups had post-treatment: fewer requests for emotional support, fewer verbal expressions of fear, fewer information-seeking questions and lower level of self-report of pain during LP.  -Painful experiences during prior LPs correlated with young age, being female, state anxiety and trait anxiety.  -Comments from children and parents indicate that children benefitted from non-pharmacologic techniques. | *Mind-body program vs control*  = for self-reported pain and distress  *Post treatment vs baseline:*  + for self-reported pain during LP | Pre-posttest design with control group, insufficient sample size, no blinding, no selective reporting, appropriate analysis, study not completed as planned (although a sample of 30 had been planned, based on a power analysis, changes in the health care delivery system during data collection greatly reduced the number of potential subjects), no missing data | Low |
| Phipps 2010 | RCT, evaluating the efficacy of **complementary therapies**, including 2 intervention groups (child-targeted or combined with parent-targeted) and 1 standard care group for somatic distress and mood disturbance associated with BMA | Children (6-18 years) with cancer undergoing stem cell BMA (n=178) | Mind-body intervention (child-targeted): based on psychoeducation, **massage and humor**  Mind-body intervention (child-targeted combined with parent-targeted): psychoeducation, **massage** and **humor** (for children) and **massage /relaxation** and **guided imagery** (for parents)  -Standard care | -Significant changes across time were observed on all patient and parent report outcomes for pain and distress  -No significant differences between treatment arms were found on pain and distress.  -No significant group differences for days in hospital, time to engraftment, or use of pharmacological interventions | *Mind-body program (child) vs mind-body program (child and parents)vs standard*  = for pain and distress, no difference between the 3 groups  *Post treatment vs baseline:*  + for pain and dis-tress (in all 3 groups) | RCT, sufficient sample size, lack of blinding, no selective reporting, correct analysis, study mostly completed as planned (some missed intervention sessions), no missing data | Moderate |
| McGrath 1986 | Pretest and posttest design, evaluating pain-management program for pain and anxiety associated with cancer treatment | Children (mean age 9 year) undergoing treatment for acute myelogenous or acute lymphoblastic leukemia (n=14) | Pain-management program: individualized, to modify expectations, control, and the relevance of the procedure. Including: **desensitization procedures, guided imagery, hypnotic like suggestions for analgesia**, relaxation training and a teaching plan. | -Children's anxiety and pain were significantly reduced at post, three-month, and six-month follow-ups | *Post treatment vs baseline:*  + for pain and anxiety at post, three-month, and six-month follow-ups | Pre-posttest design, no control group, small sample size, lack of blinding, no selective reporting, appropriate analyses, study mostly completed as planned (not discussed why 11 children did not participate in the pain-management program), no missing data | Low |
| van Aken 1986 | Observational, case series pre- and posttest, with control group, evaluating the effects of an intervention program to reduce distress during BMA | Children (mean age 8,6 years) with cancer, undergoing BMAs (n=20) | Mind-body program: including relaxation, **imagination** of a pleasant situation and arousal of the concomitant feelings, watching a model of BMA  Standard care: treatment as usual | -The experimental program was effective in reducing distress display.  -Effect of the experimental program is significant in the second phase of BMA (the phase of the punction).  -There is no significant difference in distress found between pre-procedure and post-procedure scores.  -The intensity of distress varied with age and sex of the child, was weaker in older children | *Mind-body program vs standard care*  + for distress in the second phase of BMA (the phase of the punction)  = for distress in the first phase (pre-procedure)  = for distress in the third phase (post-procedure) | Case series with control group, sufficient sample size, no randomization described, or how the choice was made for experimental or control group, no blinding, selective reporting, study mostly completed as planned, no missing data | Low |
| Broome 1992 | Multiple case study design, non-randomized, pre-post observations, evaluating the effects of distraction and **imagery** on anxiety, distress behavior and pain during LPs. | Children (3-15 years) with acute lymphocytic leukemia that previously experienced at least one LP (n=14) | Mind-body program: including **imagery**, relaxation techniques and two different **breathing techniques** | -Significant difference in pain post-treatment versus baseline  -No difference for anxiety and distress scores post-treatment versus baseline  -No difference for parent distress and anxiety post-treatment versus baseline | *Post treatment vs baseline:*  ++ for pain  = for distress  = for anxiety | Observational, pre-post design, small sample size, no control group, no randomization, no blinding, the adherence or compliance to the exercises of the intervention were not monitored, no control for attention given to the child and parents. No selective reporting, study completed as planned, no missing data | Low |
| Broome 1998 | Repeated measures, one group design evaluating the effects of relaxation, distraction and **imagery** on pain and distress associated with LPs | Children and adolescents (4-18 years) with cancer undergoing repeated LPs (n=19) | Mind-body program: child and parent were taught relaxation, distraction and **imagery**. Information package containing: a videotape of a mime demonstrating the techniques, a booklet for parents explaining how to use the techniques with their child and an age-appropriate audiotape of instructions and music to use to practice relaxation and imagery | -As compared to baseline, children reported decreased pain, but not observed behavioral distress with the intervention.  -Frequency of at-home practice was associated with greater treatment benefit; higher perceived effectiveness and frequency of practice parents’ comfort and perceived effectiveness of the techniques, were associated with decreased procedural pain  -Child temperament was related to experienced pain (between positive mood and pain)  -The majority (75%) of parents reported practicing the techniques at least monthly and rated the techniques as effective. | *Post treatment vs baseline:*  + for pain over the 5-month period  = for observed distress | Repeated measures, no control group, small sample size (although 3 centers were used, the refusal rate of 57% prevented the investigators from obtaining an adequate enough number to reach significance), lack of blinding, design controls for threats to expectancy, history, and testing, as for “spreading” the good news, selective reporting, acceptable analysis, study completed as planned, losses to follow-up with missing data | Low |
| Ahmed 2014 | Retrospective analysis , pre- post analyses to evaluate feasibility and efficacy of **Mantram meditation** for pain and distress associated with cancer treatment | Children undergoing anti-GD2 MoAb 3F8 treatment (as standard care for high-risk neuroblastoma) who received guided meditation (n=34) | **Mantram meditation:** offered to families several days a week by experienced instructors. A single specific Mantram was played on an MP3 player in the background while an experienced meditation teacher taught and led the Mantram. Mudras (hand gestures) and gentle breathing patterns (left nostril breathing, long exhalation, alternate nostril breathing) were interspersed with Mantram to help relieve tension and enhance relaxation and focus. | -No statistically significant changes after first session Mantram; however, after an average of 3 sessions, a small but significant decrease in heart rates was observed  -A significant reduction in analgesic doses was observed after the first Mantram session. Patients receiving 2 to 3 Mantram sessions consistently received fewer analgesic rescues, although no further reduction in analgesics was noted. | *Post treatment vs baseline:*  = for peak heart rate after first session Mantram)  + for peak heart rate after an average of 3 sessions  + for reduction in analgesic doses after the first (and more) Mantram session(s) | Retrospective pre-post design, no control group, sufficient sample size, no blinding. No selective reporting, study completed as planned, no missing data (the records from all patients with high-risk neuroblastoma undergoing anti-GD2 MoAb 3F8 therapy during a 10-month period were reviewed) | Low |
| **Massage** | | | | | | | |
| Post-White 2009 | RCT, crossover design in which 4 weekly **massage sessions** alternated with 4 weekly quiet-time control sessions Evaluating feasibility of providing massage to children with cancer to reduce symptoms in children associated with chemotherapy and anxiety in parents | Children (1-18 years) with cancer, received at least 2 identical cycles of chemotherapy (n=23) | **Massage therapy**: practitioner-provided. Parents’ massage: seated chair massage. Children’s massage: included back, legs, arms, stomach/chest and face. Strokes used were primarily effleurage, raking , thumb stroking and petrissage. Guided by the child’s feedback and tolerance. Very little conversation and no music was played.  Quiet Time (control condition): the child and parent participated together in the quiet-time control condition. A “do not disturb” sign was placed on the door for the same period of time as the massage. Age-appropriate toys were provided and children and parents read, rested, talked quietly, or watched a video. | -There were no significant differences between massage and quiet time for pain. Mean pain scores were low (<2.0) before and after each massage and control condition.  -Massage was more effective than quiet time at reducing heart rate in children, reducing anxiety in children less than age 14 years and reducing parent  -There were no significant changes in blood pressure, cortisol, pain, nausea, or fatigue.  -All parents reported liking their massage  -Massage in children with cancer is feasible | *Massage vs control*  = for pain  + for reducing heart rate.  + anxiety in children < 14 years  + parent anxiety | RCT, sufficient sample size, no blinding. Interview was conducted by 2 researchers who did not collect other data, interviews were transcribed verbatim and evaluated by 3 independent researchers. Some selective reporting, appropriate analysis, study not entirely completed as planned (8 male children failed to complete the study because of progressive disease, protocol changes, or their families changed their minds), some missing data | Low |
| **Music therapy** | | | | | | | |
| Pfaff 1989 | Observational pre- posttest design, evaluating the effects of **music** on pain and fear of children undergoing BMAs | Children (7-17 years) diagnosed with leukemia who have a frequency of BMAs every 6 to 8 weeks (n=9) | **Music therapy:** A relaxation master cassette containing five instrumental music selections, from which the child selected their choice of preferred music. A music therapist led the child through the music program until it was time for the BMA. The music began when the child entered the treatment room. Throughout the procedure, the music therapist coached the child on the relaxation exercises when necessary. | -As compared to baseline children in the music group had no change in experienced pain, anticipatory pain or distress | *Post treatment vs baseline:*  = for pain and distress | Observational pre-post design, no control group, insufficient sample size, no blinding, no control for attention. Some selective reporting, inappropriate analysis, study not completed as planned (3 out of 9 children did not complete the study, due to moving to another city or did not want to use music), some missing data. | Low |
| **Art therapy** | | | | | | | |
| Madden 2010 | RCT, mixed methods pilot study, repeated measures, evaluating **creative arts therapy** versus attention control for quality of life associated with chemotherapy  1.small randomized pilot with the brain tumor patients only.  2.descriptive study observed all eligible hematology/oncology patients who received creative arts therapy | 1.Children (2-18 years) receiving chemotherapy for a brain tumor (n=16)  2.Children (3-21 years) receiving chemotherapy for brain tumors and subsequently all patients receiving infusions in the outpatient hematology/oncology clinic (n=32) | **Creative arts therapy:** led by a licensed dance/movement therapist who was experienced in music and art therapies as well. The intervention consisted of 6 sessions, 2 sessions of each modality of creative arts. The sequence of activities replicated developmental expression from body movement, to sound, to graphic representation.  Attention control (volunteer’s attention): a trained volunteer sitting at the patients’ bedside in the infusion room and paying attention to them through reading, talking, or watching TV. No art activities were allowed for the control group during the volunteer’s attention. | 1. Areas that showed statistically significant improvement were: Parent-report of pain, parent report of nausea  2. As compared to baseline children in the creative arts therapy group showed improved mood, were more excited, happier and less nervous | *Creative arts therapy vs attention control*  + for self-reported pain  +parent-reported pain / nausea  *Post treatment vs baseline:*  + improved mood  + more excited, more happy, less nervous | RCT, attention control group, small sample size, randomly assigned to treatment or control group, no blinding. Some selective reporting, appropriate analysis, study not completed as planned (2 dropped out: 1 withdrawal, 1patient did not receive chemotherapy), some missing data. If the total number in a group (either creative arts or control was <4 subjects, the group was not analyzed not to bias the results. Therefore, all of the child self-report variables were eliminated. | Low |
| **Aromatherapy** | | | | | | | |
| Ndao 2012 | RCT, double-blind, placebo-controlled study, evaluating the effect of the respiratory administration of **bergamot essential oil** on anxiety, nausea, and pain during stem cell infusion. | Children (5-21 years) with malignant and non-malignant disorders undergoing stem cell transplantation (n=37) | **Bergamot essential oil**: an aromatherapy diffuser was turned on and filled or refilled with four drops of bergamot essential oil per hour.  Placebo: An aromatherapy diffuser was turned on and filled or refilled with four drops of placebo oil per hour: a non-essential oil-based scented shampoo. | -As compared to the placebo group, children in the Bergamot group reported significant more pain before transplantation and the same amount of pain compared to placebo after transplantation  -As compared to the placebo group, children in the Bergamot group showed no difference for self-reported nausea before transplantation and were more nauseous than the placebo group after transplantation  -As compared to the placebo group, children in the Bergamot group showed no difference for anxiety before transplantation and were more anxious than the placebo group after transplantation  -Although not significant, the treatment group had a higher rate of adverse events, specifically hypertension, possibly contributing to the marked differences in the experience of anxiety and nausea among the two study groups. | *Bergamot essential oil vs placebo*  *Before transplantation:*  - for self-reported pain  = for self-reported nausea  = for anxiety  *After transplantation*:  = for self-reported pain  - for self-reported nausea  - for anxiety | RCT, sufficient sample size, randomization was stratified by age and transplant type to control for the effect of different conditioning regimens, double blinded (the research assistant was blinded to treatment arm labelling and wore a mask and nose plugs upon entering the patient room to administer questionnaires and fill the diffuser. At consent, both parent and child were informed that both essential oil and placebo contained a scent, though scent type was not disclosed). No selective reporting, appropriate analysis, study not entirely completed as planned (3 randomized patients did not receive the treatment and were therefore not analyzed), no large losses to follow-up or missing data | High |

**Abbreviations**: RCT = randomized controlled trial; LP = lumbar puncture; BMA = bone marrow aspiration; IV = intravenous; CBT = cognitive-behavior therapy; GA = general anesthesia; IM = intramuscular injection

*****: studies used for GRADE assesment

**: + or - → P<0.05

++ → P<0.001

= → no significant difference

**Quality of study was evaluated based upon**

- type of study (i.e., qualitative, quantitative, and review)
- sampling strategy appropriate for research question
- method of data collection clearly described
- method of data analysis clearly described; analysis appropriate for research question
- sufficient sample size;
- blinding or data collection appropriate to study method
- appropriate analysis;
- reporting comprehensive, clearly described;
- issues with follow-up or missing data clearly described

**Used literature**

- Ahmed M, Modak S, Sequeira S: Acute pain relief after Mantram meditation in children with neuroblastoma undergoing anti-GD2 monoclonal antibody therapy. *Journal of pediatric hematology/oncology* 2014, 36(2):152-155.
- Broome ME, Lillis PP, McGahee TW, Bates T: The use of distraction and imagery with children during painful procedures. *Oncology nursing forum* 1992, 19(3):499-502.
- Broome ME, Rehwaldt M, Fogg L: Relationships between cognitive behavioral techniques, temperament, observed distress, and pain reports in children and adolescents during lumbar puncture. *Journal of pediatric nursing* 1998, 13(1):48-54.
- Hawkins P. J., Liossi C., Ewart B. W., Hatira P., Kosmidis V. H. (1998). Hypnosis in the alleviation of procedure related pain and distress in paediatric oncology patients. *Contemporary Hypnosis*, 15, 199-207. 10.1002/ch.135
- Hilgard JR, LeBaron S: Relief of anxiety and pain in children and adolescents with cancer: quantitative measures and clinical observations. *The International journal of clinical and experimental hypnosis* 1982, 30(4):417-442.
- Kellerman J, Zeltzer L, Ellenberg L, Dash J: Adolescents with cancer. Hypnosis for the reduction of the acute pain and anxiety associated with medical procedures. *Journal of adolescent health care : official publication of the Society for Adolescent Medicine* 1983, 4(2):85-90.
- Kuttner L, Bowman M, Teasdale M: Psychological treatment of distress, pain, and anxiety for young children with cancer. *Journal of developmental and behavioral pediatrics : JDBP* 1988, 9(6):374-381.
- Liossi C, Hatira P: Clinical hypnosis versus cognitive behavioral training for pain management with pediatric cancer patients undergoing bone marrow aspirations*. The International journal of clinical and experimental hypnosis* 1999, 47(2):104-116.
- Madden JR, Mowry P, Gao D, Cullen PM, Foreman NK: Creative arts therapy improves quality of life for pediatric brain tumor patients receiving outpatient chemotherapy. *Journal of pediatric oncology nursing : official journal of the Association of Pediatric Oncology Nurses* 2010, 27(3):133-145.
- McGrath PA, DeVeber LL: Helping children cope with painful procedures. *The American journal of nursing* 1986, 86(11):1278-1279.
- Ndao DH, Ladas EJ, Cheng B, Sands SA, Snyder KT, Garvin JH, Jr., Kelly KM: Inhalation aromatherapy in children and adolescents undergoing stem cell infusion: results of a placebo-controlled double-blind trial. *Psycho-oncology* 2012, 21(3):247-254.
- Pederson C: Promoting parental use of nonpharmacologic techniques with children during lumbar punctures. *Journal of pediatric oncology nursing : official journal of the Association of Pediatric Oncology Nurses* 1996, 13(1):21-30.
- Pfaff VK, Smith KE, Gowan D The effects of musicassisted refaxation on the distress of pediatric cancer patients undergoing bone marrow aspirations. *Child Health Care* 18:232-236, 1989 34.
- Phipps S., Barrera M., Vannatta K., Xiong X., Doyle J.J., Alderfer M.A.: Complementary therapies for children undergoing stem cell transplantation: Report of a multisite trial. *Cancer* 2010, 116 (16): 3924-3933
- Post-White J, Fitzgerald M, Savik K, Hooke MC, Hannahan AB, Sencer SF: Massage therapy for children with cancer. *Journal of pediatric oncology nursing : official journal of the Association of Pediatric Oncology Nurses* 2009, 26(1):16-28.
- van Aken MA, Heezen TJ, van Lieshout CF: [Bone marrow biopsy in children with leukemia. Determination and reduction of pain and fear reactions]. *Tijdschrift voor kindergeneeskunde* 1986, 54(4):112-118.
- Wall VJ, Womack W: Hypnotic versus active cognitive strategies for alleviation of procedural distress in pediatric oncology patients. *The American journal of clinical hypnosis* 1989, 31(3):181-191.
- Zeltzer L, LeBaron S: Hypnosis and nonhypnotic techniques for reduction of pain and anxiety during painful procedures in children and adolescents with cancer. *The Journal of pediatrics* 1982, 101(6):1032-1035.
