# Supplementary material for: Development of an evidence-based decision aid on complementary and alternative medicine (CAM) and pain for parents of children with cancer
Source: Support Care Cancer. 2019 Sep 6;28(5):2415–29. doi: 10.1007/s00520-019-05058-8 (PMC7083801; doi:10.1007/s00520-019-05058-8)
Supplement: Supplementary file 6 — (DOCX 28 kb) [file 520_2019_5058_MOESM6_ESM.docx]

**Online resource 6: Clinical experiences**Supportive Care in Cancer
Development of an Evidence-Based Decision Aid on Complementary and Alternative Medicine (CAM) for Parents of Children with Cancer.
Miek C. Jong, Inge Boers, Herman van Wietmarschen, Martine Busch, Marianne C. Naafs, Gert-Jan Kaspers, Wim J.E.Tissing.
Dr. Miek C. Jong, Mid Sweden University, Department of Health Sciences, Holmgatan 10, 851 70 Sundsvall, Sweden, email: miek.jong@miun.se

| **Therapy** | **Clinical experiences** | **Treatment phase** | **User** | **Type of pain** | **Uses for other complaints** | **Side effects** |
| --- | --- | --- | --- | --- | --- | --- |
| **Hypnosis, guided visualisation** | Positive | All phases, but needs attention and focus.  Better to use simple exercises during treatment phase | Children with good imagination capabilities and old enough to understand instructions | Chronic pain | General wellbeing, anxiety, depression, sleep disturbances | No side effects |
| **Massage** | Positive  Good supportive care option for parents to support their child at home  Massage technique can be adapted to age, skin sensitivity, and platelet count  Good reward after exercise therapy | All phases | Older children, no contraindication for younger children, but they rarely choose for massage or prefer massage by parents | Musculoskeletal pain, pain with anxiety and stress | General wellbeing, anxiety, depression, sleep disturbances  Good reward after exercise therapy | No side effects |
| **Breathing techniques** | Positive | All phases | From 3-4 years old and up | All types of pain | General wellbeing, sleep disturbances | No side effects |
| **Yoga, Mindful movement** | Positive  Good supportive care option in combination with physiotherapy | All phases, depending on physical condition | From 3-4 years old and up | Musculoskeletal pain, pain with anxiety and stress | General wellbeing, physical condition, fatigue, depression, improving immune system, sleep disturbances | No side effects |
| **Energy therapy** such as Reiki, Healing Touch | Positive | All phase | Children that do not want to be touched, children with a sensitive skin, children with low platelet count, children too young to participate in cognitive therapies, children not able to speak the country’s dominant language | All types of pain, especially pain with anxiety and stress or existential pain | General wellbeing, anxiety, depression, sleep disturbances | No side effects |
| **Music therapy** | Positive | All phases | All children | All types of pain, especially procedural pain | General wellbeing | No side effects |
| **Acupuncture/ acupressure** | Positive and negative (needles)  Good supportive care option for parents to use at home, | Preferably after treatment when blood cell count is restored  During treatment, techniques without needles can be used (laser or magnets) | Older children  In case children are afraid of needles, acupressure or laser can be applied | Chronic pain  and co-morbidity with other organ diseases and a complex clinical picture, neuropathic pain | General wellbeing, anxiety, sleep disturbances, continuing nausea | No side effects  Safe to use, also with low platelet count |
| **Aroma therapy** | Positive  Good supportive care option in combination with massage (eg. with peripheral neuropathy) or with energy therapies  Good supportive care option for parents to apply aromatherapy at home | All phases | Children above 2 years of age or above 20 kg | Existential pain, anxiety and stress | General wellbeing, anxiety, sleep disturbances, nausea | No side effects |
| **Biofeedback** | Positive | All phases, but needs attention and focus.  Better to use simple exercises during treatment | Age 4 years and up, old enough to understand instructions  Simple forms work best (for older children: hearth math and for younger children the bio-dot, which changes colour when they become more relaxed) | Anxiety-related pain | Anxiety, stress, nausea | No side effects |
| **Anthroposophy**  Such as rhythmic massage, etheric oils, eurythmic, art therapy and music therapy | Positive, | During treatment phase, start as early as possible | All children, specifically adolescents | All types of pain, not effective with mucositis | General wellbeing, immune system support, physical conditions and fatigue | No side effects |
